# KPNB1 inhibition disrupts proteostasis and triggers unfolded protein response-mediated apoptosis in glioblastoma cells

**DOI:** 10.1038/s41388-018-0180-9

**Published:** 2018-03-09

**Authors:** Zhi-Chuan Zhu, Ji-Wei Liu, Kui Li, Jing Zheng, Zhi-Qi Xiong

**Affiliations:** 1 Institute of Neuroscience, State Key Laboratory of Neuroscience, CAS Center for Excellence in Brain Science and Intelligence Technology, Chinese Academy of Sciences, 200031 Shanghai, China; 2 School of Pharmacy, East China University of Science and Technology, 200237 Shanghai, China; 3 University of Chinese Academy of Sciences, 100049 Beijing, China; 4 School of Life Science and Technology, ShanghaiTech University, 201210 Shanghai, China

## Abstract

The nuclear import receptor karyopherin β1 (KPNB1) is involved in the nuclear import of most proteins and in the regulation of multiple mitotic events. Upregulation of KPNB1 has been observed in cancers including glioblastoma. Depletion of KPNB1 induces mitotic arrest and apoptosis in cancer cells, but the underlying mechanism is not clearly elucidated. Here, we found that downregulation and functional inhibition of KPNB1 in glioblastoma cells induced growth arrest and apoptosis without apparent mitotic arrest. KPNB1 inhibition upregulated Puma and Noxa and freed Mcl-1-sequestered Bax and Bak, leading to mitochondrial outer membrane permeabilization (MOMP) and apoptosis. Moreover, combination of Bcl-xL inhibitors and KPNB1 inhibition enhanced apoptosis in glioblastoma cells. KPNB1 inhibition promoted cytosolic retention of its cargo and impaired cellular proteostasis, resulting in elevated polyubiquitination, formation of aggresome-like-induced structure (ALIS), and unfolded protein response (UPR). Ubiquitination elevation and UPR activation in KPNB1-deficient cells were reversed by KPNB1 overexpression or inhibitors of protein synthesis but aggravated by inhibitors of autophagy-lysosome or proteasome, indicating that rebalance of cytosolic/nuclear protein distribution and alleviation of protein overload favor proteostasis and cell survival. Chronic activation of eIF2α/ATF4 cascade of UPR was responsible for the upregulation of Puma and Noxa, apoptosis and ABT-263 sensitivity. Taken together, our findings demonstrate that KPNB1 is required for proteostasis maintenance and its inhibition induces apoptosis in glioblastoma cells through UPR-mediated deregulation of Bcl-2 family members.

## Introduction

Karyopherin β1 (KPNB1), also known as importin β, is a nuclear transport receptor belonging to the karyopherin family that is involved in transporting proteins through the nuclear pore [1]. KPNB1 contains a C-terminal region that interacts with the importin β binding domain of KPNAs (another subfamily of karyopherin proteins that bind cargos and link them to KPNB1), a central region that interacts with FxFG repeats of nucleoporins and an N-terminal region that interacts with RanGTP [2]. Generally, KPNB1 transports cargos from the cytosol to nucleus through nuclear pore complexes using KPNAs as adapters or by directly interacting with cargos where KPNAs acts as binding competitors. After translocation with cargos from the cytosol to nucleus, RanGTP binds to KPNB1 to let cargos free from KPNB1. The concentration difference of RanGTP between the nucleus and cytosol ensures that cargos captured by KPNB1 in the cytosol gets released in the nucleus to become active [3]. In addition to nuclear import, KPNB1 also functions in mitosis, including mitotic spindle assembly, microtubule-kinetochore attachment, mitotic exit, and nuclear envelop assembly [3–8].

KPNB1 concentration correlates with its nuclear import efficiency and speed [9]. Many KPNB1 cargos are critical for tumorigenesis, including core signaling transducers (STAT3, NF-κB p65, Gli1), growth factor receptors (ErbB-2, EGFR, c-Met), death receptors (DR5), actin modulation protein (CapG), and transcriptional factors (Snail) [10–18]. The nuclear localization of these cargos is required for their roles in tumorigenesis. Consistently, upregulation of KPNB1 expression has been observed in various cancers. In cancers, KPNB1 expression is regulated by EZH2-miR-30d axis and E2F, while KPNB1-mediated nuclear import is inhibited by p53-induced factor Ei24 [19–21]. KPNB1 knockdown in cervical cancer cells inhibits cell growth by inducing prolonged mitotic arrest and apoptosis. This apoptotic effect might be mediated by downregulation and Noxa-associated inactivation of Mcl-1 [22]. KPNB1 expression is required for NF-κB p65 nuclear import and tumor progression in multiple myeloma, hepatocellular carcinoma, and diffuse large B-cell lymphoma. However, whether p65 nuclear import mediates the pro-oncogenic function of KPNB1 in these cancers has not been validated [23–25]. Collectively, the susceptibility of cancer cells to KPNB1 deficiency-induced apoptosis makes KPNB1 a candidate target for cancer therapy [22, 23, 26].

Glioblastoma multiforme (GBM) is the most common malignant brain tumor in adults and remains incurable using current therapies, which urgently needs deeper understanding of its molecular pathology to develop novel therapeutic strategies. In this study, we show that KPNB1 is required for glioblastoma survival. KPNB1 deficiency disturbed proteostasis, caused UPR-mediated deregulation of Bcl-2 family proteins, and ultimately induced apoptosis, which can be potentiated by Bcl-xL inhibitors, lysosome inhibitors or proteasome inhibitors. These data can have translational implication in glioblastoma treatment.

## Results

### Depletion of KPNB1 inhibits viability in glioblastoma cells

As reported by the REMBTANDT knowledgebase (http://www.betastasis.com/glioma/rembrandt/) [27], *KPNB1* mRNA expression in GBM samples is higher than that in normal brain samples (Supplementary Fig. 1a). Furthermore, the Kaplan–Meier curves revealed significant differences in survival for KPNB1, with the higher expression having the poorer survival, not only in all glioma samples, but also the GBM sample subset (Supplementary Fig. 1b). Therefore, *KPNB1* may play a role in the progression of glioblastomas.

**Fig. 1:**
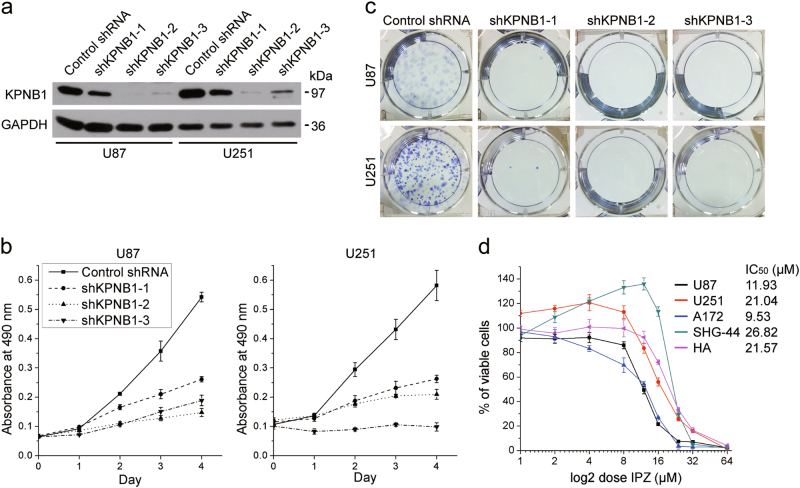
KPNB1 deficiency inhibits glioblastoma cell viability. **a** U87 and U251 cells were infected with lentiviruses encoding either a scrambled shRNA (Control shRNA) or KPNB1-specific shRNAs (shKPNB1-1, 2, and 3) to generate KPNB1 knockdown glioma cell lines. KPNB1 knockdown was confirmed by western blots. GAPDH was used as the loading control. **b** Proliferation rates of U87 and U251 cells treated as in **a** were measured by MTT assays (*n* = 5, mean ± s.d.). **c** Colony formation of U87 and U251 treated as in **a** were measured by colony formation assays (*n* = 3). Representitive images were chosen from three independent experiments. **d** U87, U251, A172, SHG-44, and HA cells were treated with indicated concentration of IPZ for 48 h, followed by MTT assay (*n* = 5, mean ± s.d.). Half maximal inhibitory concentrations (IC_50_s) of cells were listed on the top-right panel

To investigate this point, we depleted KPNB1 using three short hairpin RNA (shRNA) constructs (shKPNB1-1, 2, and 3) targeting human KPNB1 in U87 and U251 glioblastoma cells (Fig. 1a). KPNB1 knockdown inhibited cell proliferation and colony formation (Fig. 1b, c). We further investigated the effect of importazole (IPZ), a small molecule that inhibits KPNB1-mediated protein nuclear import [28], in four glioblastoma cell lines (U87, U251, A172, and SHG-44) and human fetal astrocytes (HA). IC_50_ values of IPZ in U87 and A172 (11.95 and 9.53 µM, respectively) were lower than those of U251, SHG-44, and HA (21.04, 26.82, and 21.57 µM, respectively) (Fig. 1d). However, HA here could not represent mature astrocytes because levels of KPNB1 and glioblastoma stem cell markers Oct4 and CD133 in HA was similar to those in U87 and U251 (Supplementary Fig. 2). Together, these findings suggest that knockdown or pharmacological inhibition of KPNB1 suppresses glioblastoma cell viability.

### KPNB1 inhibition induces apoptosis in glioblastoma cells

**Fig. 2:**
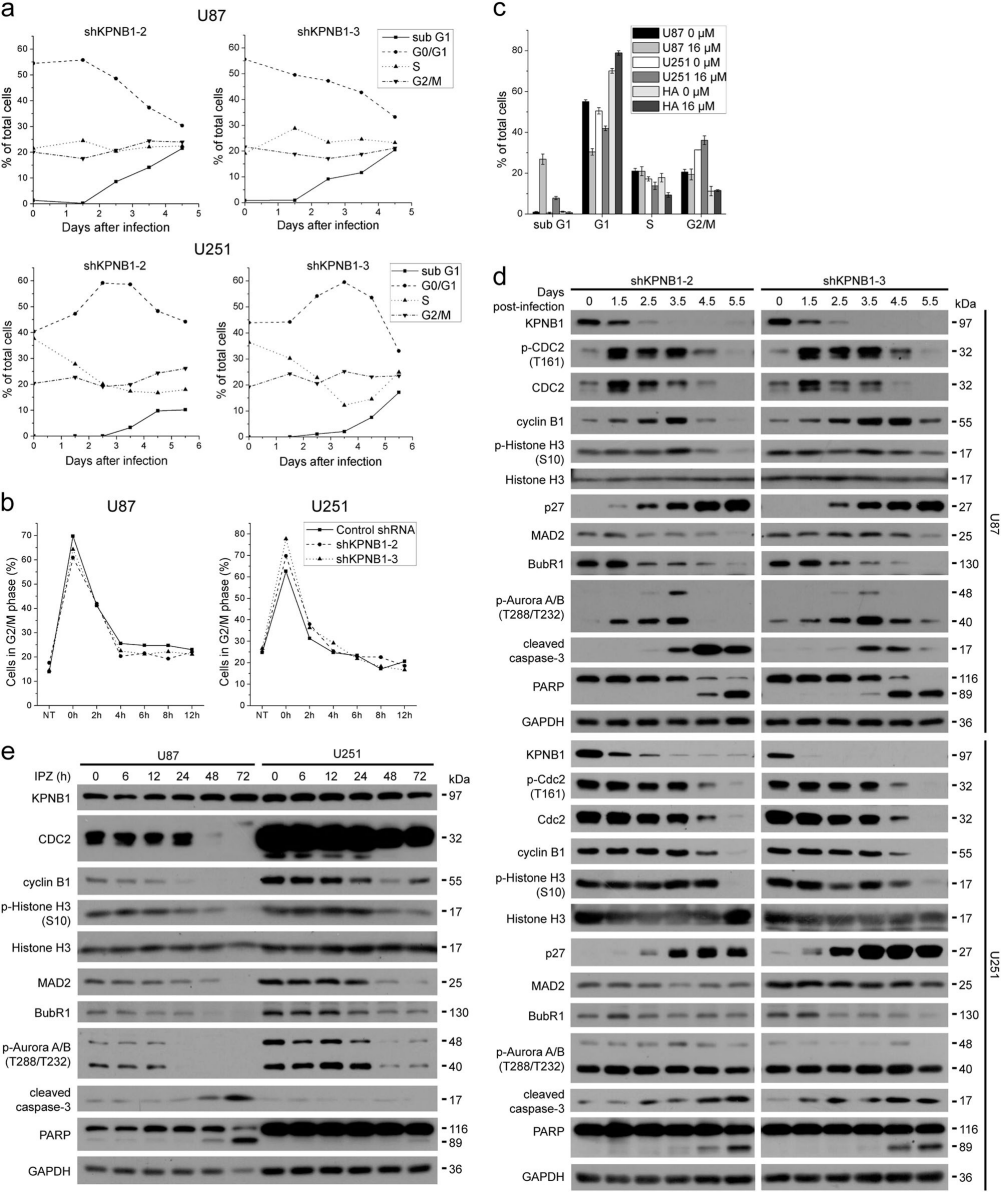
KPNB1 deficiency induces apoptosis in glioblastoma cells. **a** U87 and U251 cells infected with lentiviruses encoding shKPNB1s were harvested at various time points, followed by cell cycle distribution analysis. **b** Control shRNA and shKPNB1s-U87 and U251 cells were synchronized in mitosis following thymidine-nocodazole block (after 21 h of infection, cells were in turn treated with 2 mM thymidine for 24 h, fresh medium for 3 h, and 100 ng/ml nocodazole for 12 h) [22], then released and harvested at indicated time points. Percentages of G2/M population were analyzed by flow cytometry. NT not treated. **c** The cell cycle distribution of U87, U251, and HA cells treated with IPZ (16 μM) for 3 days were measured by flow cytometry (*n* = 3, mean ± s.d.). **d**, **e** U87 and U251 infected with shKPNB1-encoding lentiviruses (**d**) or treated with IPZ (16 μM) (**e**) were harvested at indicated time points. Levels of proteins associated with mitosis, G1 phase arrest, spindle assembly checkpoint, and apoptosis were analyzed by western blots. GAPDH was used as the loading control

KPNB1 regulates multiple stages of mitosis and inhibition of KPNB1 causes mitotic arrest in cervical cancer cells [22]. To dissect the role of KPNB1 in the cell cycle regulation, we first generated KPNB1-deficient U87 and U251 cells by the infection of shKPNB1s-encoding lentiviruses. Our results showed that KPNB1 knockdown induced the accumulation of the sub-G1 cell population which indicates cell death and concurrently decreased the G1 population. In contrast, KPNB1 knockdown did not change the G2/M population (Fig. 2a). Furthermore, KPNB1 knockdown did not change the mitotic exit of cells released from mitotic block (Fig. 2b), suggesting that KPNB1 knockdown did not cause mitotic arrest in glioblastoma cells. Similar to KPNB1 knockdown, functional inhibition of KPNB1 using IPZ in U87 and U251 cells also increased the sub-G1 population and decreased the G1 population, with the G2/M population barely changed. In comparison, IPZ treatment of HA did not induce cell death, but increased the G1 population instead (Fig. 2c). These results suggest that KPNB1 inhibition induces cell death, but not mitotic arrest in glioblastoma cells.

We next analyzed levels of proteins associated with mitosis (p-CDC2, cyclin B1, p-Aurora A/B, p-histone H3), G1 phase arrest (p27), spindle assembly checkpoint (SAC) (MAD2, BubR1), and apoptosis (cleaved caspase-3, PARP) in U87 and U251 cells infected by shKPNB1-encoding lentiviruses in a time-dependent manner (Fig. 2d). Our results showed that at early stages (infection after 0–3.5 days), upregulation of mitosis markers p-CDC2 (T161), CDC2, cyclin B1, and p-Aurora A (T288)/Aurora B (T232) was observed in U87 cells, but not in U251 cells. After 4.5 days infection, expressions of all mitosis markers dramatically decreased and were barely detected after 5.5 days infection in both U87 and U251 cells, suggesting the occurrence of abnormal mitosis. The level of p27 was increased in a time-dependent manner in both cell lines, suggesting the induction of G1 phase arrest. During mitosis, SAC remains active to ensure proper formation of mitotic spindle and kinetochore-microtubule attachment, resulting in elevated expression of cyclin B1 and sustained activity of CDC2. Our results showed that after infection, dissipation of BubR1 was observed before the culminating of cyclin B1 level, whereas dissipation of MAD2 was lagged and moderate, suggesting that SAC could not be sustainably activated. Consistent with our above findings, cleavage of caspase-3 and PARP was induced after infection, indicating the onset of apoptosis. Similar to KPNB1 knockdown, treatment of U87 and U251 cells with IPZ for 48 h dramatically downregulated mitotic markers and SAC-associated proteins and upregulated apoptosis markers (Fig. 2e). Taken together, our results suggest that inhibition of KPNB1 in glioblastoma cells induced apoptosis and abnormal mitosis, but not the mitotic arrest due to weak SAC activity.

### Deregulation of Bcl-2 proteins mediates KPNB1 inhibition-induced apoptosis in glioblastoma cells

**Fig. 3:**
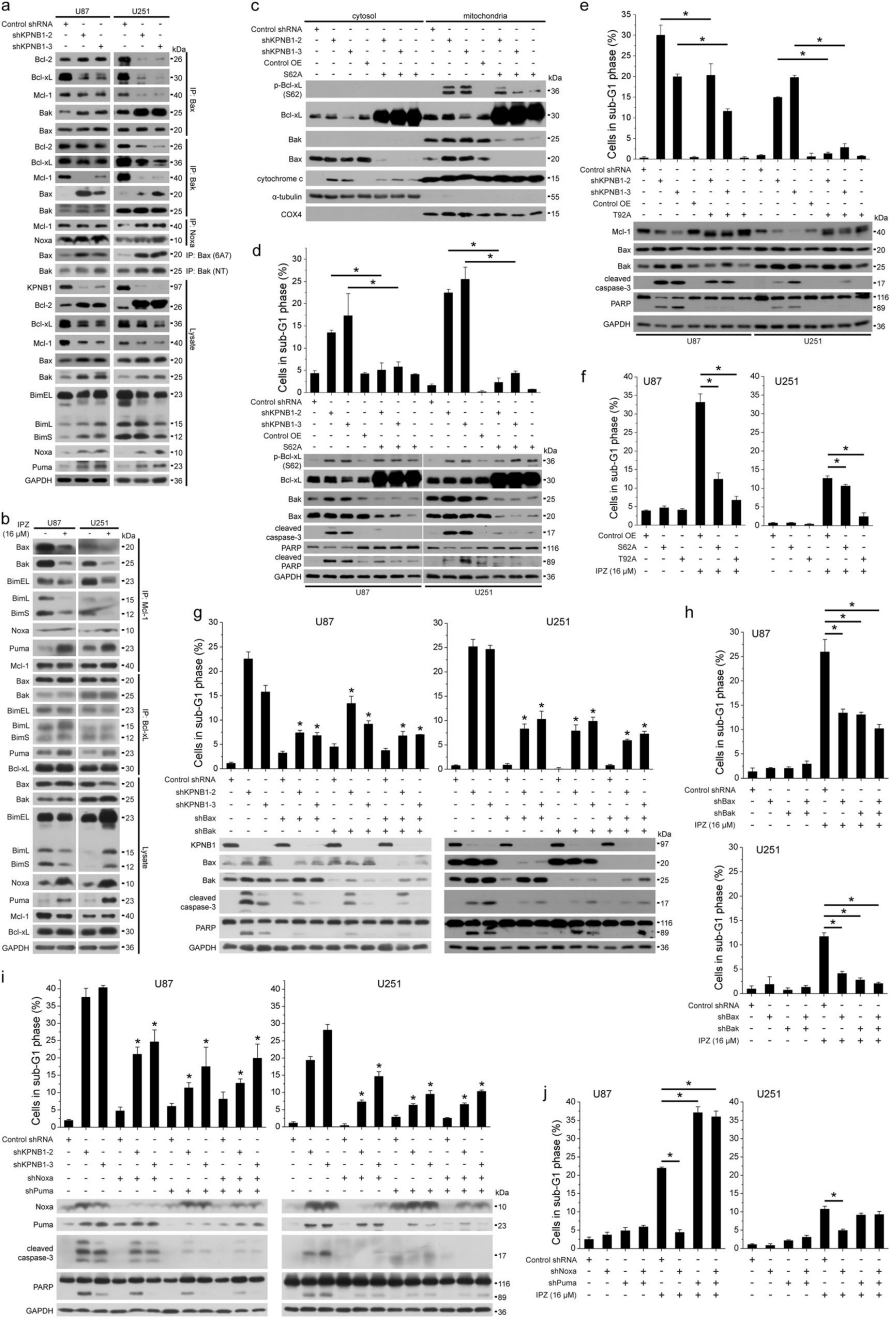
Deregulation of Bcl-2 proteins mediates KPNB1 inhibition-induced apoptosis in glioblastoma cells. **a** Total Bax, active Bax, total Bak, and active Bak were immunoprecipitated from control and shKPNB1-U87 and U251 cells, respectively. Interaction proteins were identified by western blots. **b** Mcl-1 and Bcl-xL were immunoprecipitated from IPZ (16 μM)-treated U87 and U251 cells, followed by western blots. **c**, **d** U87 and U251 cells were infected with lentiviruses encoding shKPNB1s and/or Bcl-xL (S62A). **c** Western blots analysis of the cytosolic and mitochondrial proteins in U87 cells. **d** The sub-G1 population analysis (*n* = 2, mean ± s.d., *P < 0.05) and western blots in U87 and U251 cells. **e** U87 and U251 cells were infected with lentiviruses encoding shKPNB1s and/or Mcl-1 (T92A), followed by the sub-G1 population analysis (*n* = 2, mean ± s.d., *P < 0.05) and western blots. **f** U87 and U251 cells expressing Bcl-xL (S62A) or Mcl-1 (T92A) were treated with IPZ (16 μM), followed by sub-G1 population analysis (*n* = 2, mean ± s.d., *P < 0.05). **g** U87 and U251 cells were infected with lentiviruses as indicated, followed by the sub-G1 population analysis (*n* = 2, mean ± s.d., *P < 0.05, compared with respective shKPNB1 group) and western blots. **h** U87 and U251 cells expressing shBax and/or shBak were treated with IPZ (16 μM), followed by sub-G1 population analysis (*n* = 3, mean ± s.d., *P < 0.05). **i** U87 and U251 cells were infected with lentiviruses as indicated, followed by the sub-G1 population analysis (*n* = 3, mean ± s.d., *P < 0.05, compared with respective shKPNB1 group) and western blots. **j** U87 and U251 cells expressing shNoxa and/or shPuma were treated with IPZ (16 μM), followed by sub-G1 population analysis (*n* = 3, mean ± s.d., *P < 0.05). For western blots, GAPDH, α-tubulin, and COX4 were used as the loading control of total cell lysate, cytosol fraction, and mitochondria fraction, respectively

As KPNB1 inhibition triggered apoptosis in U87 and U251 cells, we investigated the mitochondrial signaling of apoptosis, including three subgroups of Bcl-2 protein family that control the integrity of mitochondrial outer membrane: anti-apoptotic proteins Bcl-2, Bcl-xL, and Mcl-1, which bind and prohibit the activation of Bax and Bak; pro-apoptotic proteins Bax and Bak, whose activation triggers MOMP; BH3-only proteins Bim, Noxa, and Puma, which bind anti-apoptotic proteins to free Bax and Bak [29]. We found that both KPNB1 knockdown and IPZ treatment in U87 and U251 cells downregulated Mcl-1 (except in IPZ-treated U251 cells) and upregulated Bak, Noxa, Puma (Fig. 3a, b). In addition, KPNB1 knockdown prevented interactions between anti-apoptotic proteins (Bcl-xL, Mcl-1) and pro-apoptotic proteins (Bax, Bak) and promoted heterodimerization and activation of Bax and Bak (Fig. 3a), whereas IPZ treatment enhanced Noxa/Mcl-1, Puma/Mcl-1, and Puma/Bcl-xL interaction but only prevented bindings of Mcl-1 rather than Bcl-xL to Bax, Bak, and Bim (Fig. 3b). Noxa/Mcl-1 interaction was enhanced in U251 cells upon KPNB1 knockdown (Fig. 3a). Accumulation of active Bax and Bak eventually triggered MOMP, the decisive event of mitochondrial apoptosis, in U87 and U251 cells following KPNB1 knockdown or IPZ treatment (Supplementary Fig. 3). Moreover, KPNB1 knockdown in U87 cells accumulated Bax and the non-functional phosphorylated Bcl-xL (S62) [30] in the mitochondria and triggered cytosolic leakage of cytochrome c (Fig. 3c). Overexpression of the phospho-defective mutant Bcl-xL (S62A) downregulated Bax and Bak, reversed the mitochondrial translocation of Bax, and rescued the cytosolic leakage of cytochrome c and apoptosis induced by KPNB1 knockdown (Fig. 3d). Modest ectopic expression of non-degradable phospho-defective mutant Mcl-1 (T92A) also rescued apoptosis induced by KPNB1 knockdown (Fig. 3e). Rescue of IPZ-triggered apoptosis was more thorough with Mcl-1 (T92A) overexpression than Bcl-xL (S62A) overexpression (Fig. 3f), suggesting that IPZ-triggered apoptosis was more dependent on the neutralization of Mcl-1 than Bcl-xL. Depletion of Bax and Bak partially rescued apoptosis induced by KPNB1 knockdown or IPZ treatment (Fig. 3g, h). In addition, KPNB1 knockdown-induced apoptosis was partially rescued by depletion of Noxa and Puma, while IPZ-induced apoptosis was rescued by Noxa rather than Puma depletion (Fig. 3I, j). ShPuma disturbed the knockdown efficacy and anti-apoptotic effect of shNoxa probably by compensatory mechanism. Together, these results suggest that KPNB1 inhibition downregulates Mcl-1, upregulates Bak, Puma and Noxa, and disrupts bindings of Mcl-1 to Bax and Bak, thus leading to apoptosis.

### ABT-263 enhances KPNB1 inhibition-induced apoptosis in a Bcl-xL/Mcl-1-dependent manner

**Fig. 4:**
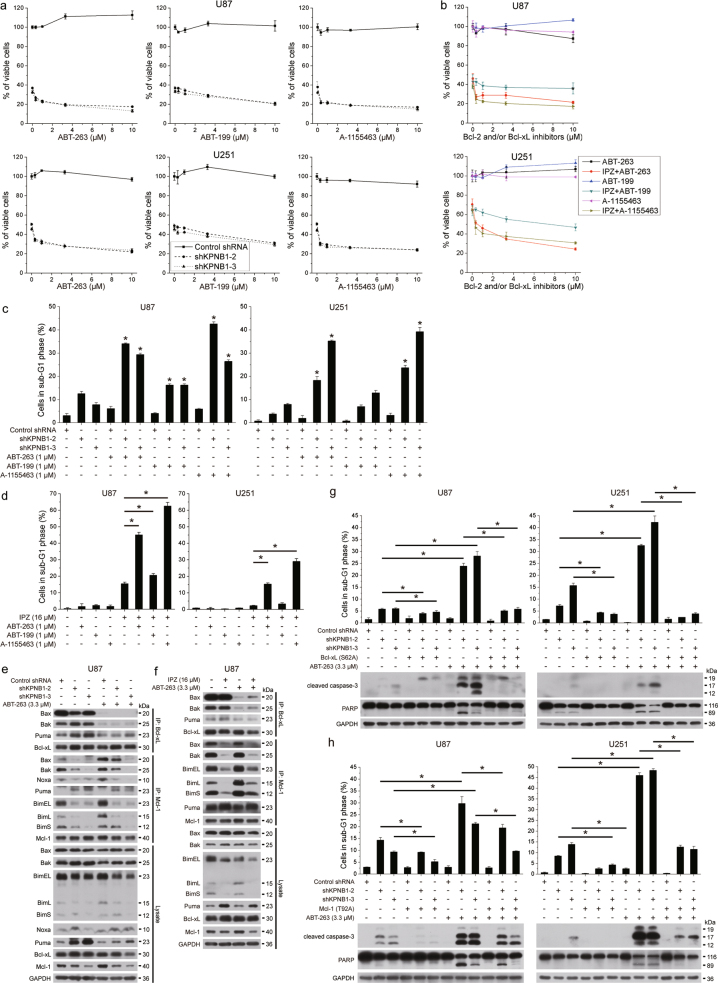
ABT-263 enhances KPNB1 inhibition-induced apoptosis in a Bcl-xL/Mcl-1-dependent manner. **a,** **b** U87 and U251 cells expressing shKPNB1s (**a**) or pre-treated with IPZ (**b**) were incubated with ABT-263, ABT-199, or A-1155463 at indicated concentration for 48 h. Cell viabilities were measured by MTT assays (*n* = 3, mean ± s.d.). **c**, **d** U87 and U251 cells expressing shKPNB1s (**c**) or pre-treated with IPZ (**d**) were incubated with ABT-263, ABT-199, or A-1155463 (1 μM) for 24 h. The sub-G1 population was measured by flow cytometry (*n* = 2, mean ± s.d., *P < 0.05, compared with respective shKPNB1 group). **e,** **f** U87 cells expressing shKPNB1s (**e**) or pre-treated with IPZ (**f**) were incubated with ABT-263 (3.3 μM) for 24 h. Bcl-xL or Mcl-1 was immunoprecipitated, followed by western blots. **g**, **h** U87 and U251 cells expressing shKPNB1s along with Bcl-xL (S62A) (**g**) or Mcl-1 (T92A) (**h**) were treated with ABT-263 (3.3 μM) for 24 h, followed by western blots and sub-G1 population analysis (*n* = 2, mean ± s.d., *P < 0.05). For western blots, GAPDH was used as the loading control

Bcl-xL knockdown in KPNB1-deficient U87 and U251 cells enhanced apoptosis (Supplementary Fig. 4). Thus, we hypothesized that Bcl-xL inhibitors can enhance apoptosis in shKPNB1-expressing or IPZ-treated glioblastoma cells. To test this hypothesis, shKPNB1 and control cells were treated with Bcl-2/Bcl-xL inhibitor ABT-263, Bcl-xL-specific inhibitor A-1155463, and Bcl-2-specific inhibitor ABT-199, respectively. Our results showed that KPNB1 knockdown sensitized U87 and U251 cells to inhibitors-induced viability inhibition and apoptosis (Fig. 4a, c). Whereas shKPNB1 cells responded to as low as 0.33 μM ABT-263 and A-1155463, control cells remained unresponsive to 10 μM ABT-263 and A-1155463 (Fig. 4a). Furthermore, A-1155463-induced viability inhibition and apoptosis were similar to ABT-263, but greater than ABT-199 (Fig. 4a, c), suggesting that Bcl-xL was the major player for the apoptosis enhancement of ABT-263 in shKPNB1-expressing cells. Similar results were obtained in IPZ-treated U87 and U251 cells (Fig. 4b, d). On the molecular level, ABT-263 treatment abolished bindings of Bcl-xL to Bax and Bak in both control and KPNB1-deficient U87 cells (Fig. 4e, f). However, compared with control cells, KPNB1-deficient U87 cells have enhanced Mcl-1/Puma interaction and attenuated bindings of Mcl-1 to Bax, Bak, and Bim, thus losing ABT-263 resistance (Fig. 4e, f). Overexpression of Bcl-xL (S62A) or Mcl-1 (T92A) mutants partially rescued ABT-263-enhanced apoptosis in shKPNB1 U87 and U251 cells (Fig. 4g, h), suggesting that KPNB1 deficiency sensitizes glioblastoma cells to ABT-263 in a Bcl-xL/Mcl-1-dependent manner.

### Proteostasis perturbation and UPR upon KPNB1 inhibition dictate glioblastoma cell apoptosis

**Fig. 5:**
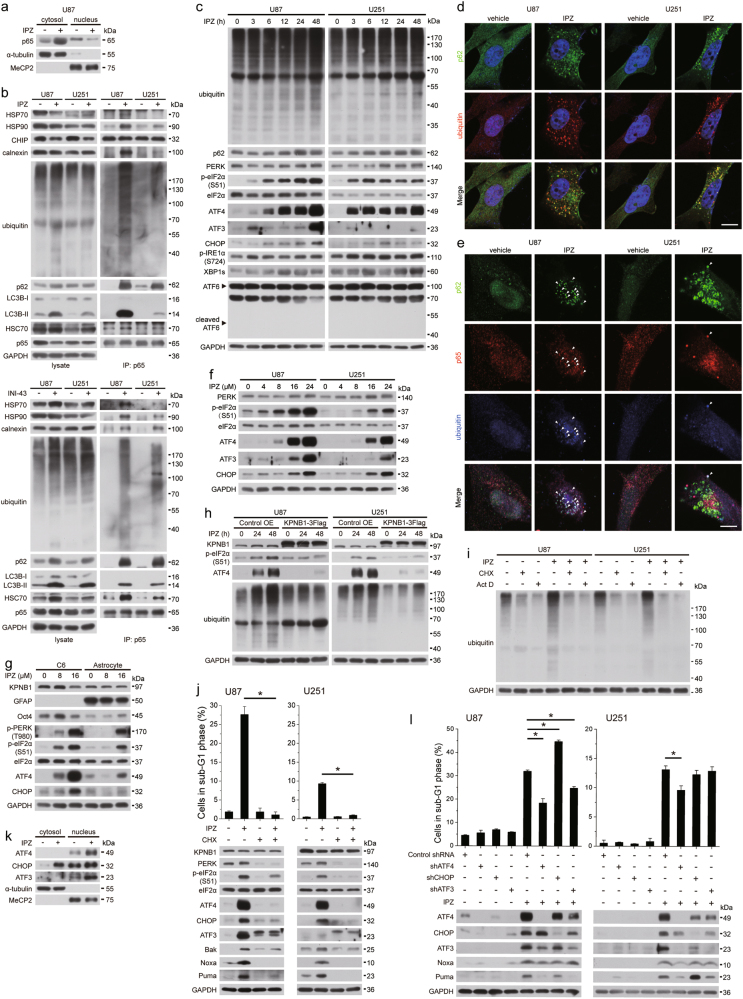
Proteostasis perturbation and UPR upon KPNB1 inhibition dictate glioblastoma cell apoptosis. **a** Western blots analysis of cytosolic and nuclear p65 level in IPZ (16 μM)-treated U87 cells. **b** p65 was immunoprecipitated from IPZ (16 μM)-treated or INI-43 (8 μM)-treated U87 and U251 cells, followed by western blots. **c** U87 and U251 treated with IPZ (16 μM) were harvested at indicated time points before western blots. **d** Representative images showing p62 and ubiquitin staining in IPZ (16 μM)-treated U87 and U251 cells. Magnification, ×60; scale bar, 10 μm. **e** Representative images showing p62, p65, and ubiquitin staining in IPZ (16 μM)-treated U87 and U251 cells. Arrows indicate colocalization of p62, p65, and ubiquitin. Magnification, ×60; scale bar, 10 μm. **f** U87 and U251 cells were treated with IPZ at indicated concentrations for 24 h and subjected to western blots. **g** C6 cells and rat astrocytes were treated with IPZ at indicated concentrations for 48 h and subjected to western blots. **h** U87 and U251 cells overexpressing KPNB1-3Flag were treated with IPZ (16 μM) for indicated times and subjected to western blots. **i** U87 and U251 cells treated with IPZ (16 μM) were treated with Act D (0.5 μg/ml) or CHX (U87: 0.5 μg/ml and U251: 2.5 μg/ml) and subjected to western blots. **j** U87 and U251 cells were treated with or without IPZ (16 μM) and CHX and subjected to flow cytometry (*n* = 2, mean ± s.d., *P < 0.05) and western blots. **k** Western blots analysis of cytosolic and nuclear levels of ATF4, CHOP, and ATF3 in IPZ (16 μM)-treated U87 cells. **l** U87 and U251 cells expressing shATF4, shCHOP, or shATF3 were treated with IPZ (16 μM) and subjected to flow cytometry (*n* = 2, mean ± s.d., *P < 0.05) and western blots. For western blots, α-tubulin, MeCP2, and GAPDH were used as the loading control of cytosol fraction, mitochondria fraction, and total cell lysate, respectively

To explore the mechanism linking deregulation of Bcl-2 proteins to KPNB1 inhibition, we investigated the nuclear import function of KPNB1. As KPNB1 mediates the classic nuclear import pathway, KPNB1 inhibition might result in the cytosolic accumulation of a substantial number of proteins should have localized in the nucleus, leading to cytosolic protein overload. We exemplified this hypothesis with KPNB1’s cargo p65. We found that KPNB1 inhibition promoted the cytosolic accumulation of p65 in U87 cells (Fig. 5a and Supplementary Fig. 5a). Polyubiquitination of both p65 and total proteins were upregulated in U87 and U251 cells following KPNB1 inhibition (Fig. 5b, c and Supplementary Fig. 5b, c). KPNB1 inhibition also upregulated p62 (Fig. 5b, c and Supplementary Fig. 5c), an ubiquitin receptor-mediating protein degradation through the macroautophagy or proteasome to maintain proteostasis under stress conditions, and induced the formation of ALIS which indicates cellular adjustment to perturbed proteostasis [31], as p62 and ubiquitin aggregated and colocalized in the cytosol (Fig. 5d). IPZ treatment promoted bindings of p62 and the autophagic receptor LC3B-II to p65 and triple colocalization of p62, p65, and ubiquitin, whose levels correlated with that of ubiquitinated p65 and IPZ sensitivity (Fig. 5b, e), indicating that p62 recruits the autophagosome membrane to ubiquitinated p65 aggregates. Albeit some p65 aggregates colocalized with ubiquitin, colocalization of p62, p65, and ubiquitin was much less in U251 cells than that in U87 cells (Fig. 5e), suggesting proteins other than p62 regulate ubiquitinated p65 aggregates. IPZ treatment also promoted bindings of HSP70, HSP90, HSC70, and calnexin to p65, whose levels correlated with the ubiquitinated p65 level (Fig. 5b). The HSP90 inhibitor 17-AAG potentiated IPZ-induced viability inhibition in U87 cells with relatively high HSP90/p65 interaction (Supplementary Fig. 5d). P65/CHIP (C-terminus of HSC70-interacting protein) interaction was unchanged upon IPZ treatment (Fig. 5b), precluding the involvement of HSP70/CHIP in p65 degradation. These indicate the involvement of chaperones of quality control systems in regulating excessive KPNB1 cargos and adaption to stresses. Above results suggest that KPNB1-deficient cells utilize protein degradation pathways and quality control systems to defend against protein overload. Protein overload might also cause endoplasmic reticulum (ER) stress response and activate UPR which inhibit protein translation to retain cellular proteostasis [32]. In U87 and U251 cells, KPNB1 knockdown or IPZ treatment activated PERK branch of UPR, as revealed by the upregulation of PERK, phosphorylated eIF2α (S51), ATF4, ATF3 and CHOP, and IRE1α branch of UPR, as revealed by the upregulation of phosphorylated IRE1α (S724) and XBP1s, but not ATF6 branch of UPR, as ATF6 was not cleaved (Fig. 5c, f and Supplementary Fig. 5c). These indicate the induction of UPR in glioblastoma cells. In rat glial system, IPZ showed selectivity in PERK signaling activation and viability inhibition in rat glioblastoma cell line C6 when compared with rat primary astrocytes (Fig. 5g and Supplementary Fig. 5e). Restoring nuclear import by KPNB1 overexpression prevented IPZ-induced protein ubiquitination and PERK signaling activation in U87 and U251 cells (Fig. 5h), suggesting that proper cytosolic/nuclear protein ratio is required for proteostasis. Partial inhibition of protein synthesis by low dose of the translation inhibitor cycloheximide (CHX) prevented protein ubiquitnation, PERK signaling activation, upregulation of pro-apoptotic Bcl-2 proteins, and apoptosis induced by IPZ treatment or KPNB1 knockdown in U87 and U251 cells (Fig. 5i, j and Supplementary Fig. 5f, g). Low dose of the transcription inhibitor actinomycin D (Act D) had similar reversing effect on protein ubiquitination (Fig. 5i and Supplementary Fig. 5f). PERK branch is usually responsible for UPR-induced apoptosis via the effect of transcriptional factors ATF4, CHOP, and ATF3, whereas IRE1α branch primarily regulates adaptive/survival response [32]. However, KPNB1 inhibition detained CHOP in the cytosol and suppressed its nuclear accumulation probably abolishing its function (Fig. 5k and Supplementary Fig. 5h). To prove that KPNB1 deficiency-induced apoptosis in U87 and U251 cells is dependent on PERK signaling, we knocked down ATF4, CHOP, and ATF3, respectively. ATF4 depletion, not CHOP or ATF3 depletion, partially reversed apoptosis and upregulation of Noxa and Puma induced by KPNB1 inhibition (Fig. 5l and Supplementary Fig. 5i), which favors our hypothesis. These suggest that reducing total protein amount in KPNB1-deficient glioblastoma cells by translation inhibition via mechanisms like ER stress aims at restoring proteostasis, whereas chronic ER stress upregulates pro-apoptotic Bcl-2 proteins and induces apoptosis. Together, these results suggest that KPNB1 deprivation in glioblastoma cells impairs protein nuclear import and perturbs proteostasis, which govern the intensity of UPR and apoptosis.

### Autophagy-lysosome-mediated and proteasome-mediated protein degradation promotes survival of KPNB1-deficient glioblastoma cells

**Fig. 6:**
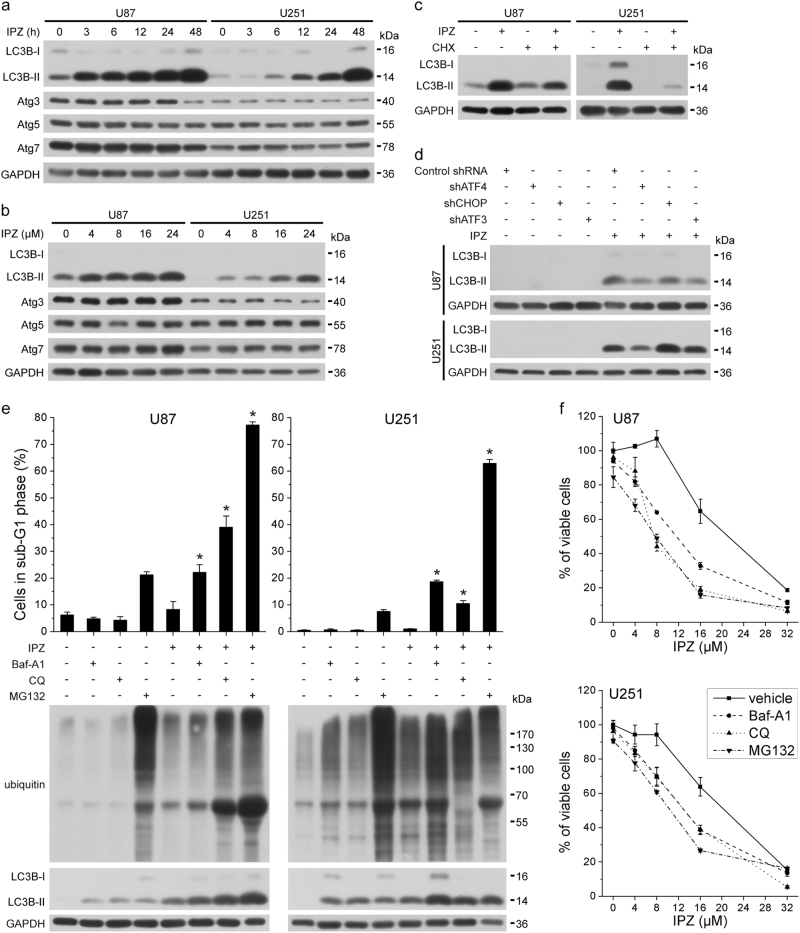
Lysosome and proteasome-mediated protein degradation promotes survival of IPZ-treated glioblastoma cells. **a** U87 and U251 cells treated with IPZ (16 μM) were harvested at indicated times, followed by western blots analysis for autophagy-associated proteins. **b** U87 and U251 cells treated with IPZ (16 μM) at indicated concentrations for 24 h, followed by western blot analysis for autophagy-associated proteins. **c** LC3B levels in U87 and U251 cells treated with IPZ (16 μM) and/or CHX (U87: 0.5 μg/ml and U251: 2.5 μg/ml). **d** U87 and U251 cells expressing shATF4, shCHOP, or shATF3 were treated with IPZ (16 μM), followed by LC3B levels analysis. **e** IPZ-treated U87 and U251 cells were treated with or without Baf-A1 (5 nM), CQ (40 μM), or MG132 (1 μM) for 48 h, followed by flow cytometry (*n* = 3, mean ± s.d., *P < 0.05, compared with IPZ) and western blots. **f** The cell viability of U87 and U251 cells treated as in **e** was measured by MTT (*n* = 3, mean ± s.d.). For western blots, GAPDH was used as the loading control

Autophagy is concurrent with apoptosis under ER stress [33]. Above results have shown that the KPNB1 cargo p65 interacted with the autophagy marker LC3B-II in IPZ-treated U87 and U251 cells. Here, we found that IPZ treatment induced time-dependent and dose-dependent upregulation of LC3B-II, but not other autophagy regulators like Atg3, Atg5, and Atg7 (Fig. 6a, b). IPZ-induced LC3B upregulation was reversed by CHX or ATF4 knockdown (Fig. 6c, d), suggesting that IPZ-induced autophagy is regulated by proteostasis and UPR. Degradation of aberrant proteins depends on the autophagy-lysosome and proteasome pathway [34]. To assess the contribution of protein degradation pathways to proteostasis, lysosome (autophagic flux) inhibitors Bafilomycin A1 (Baf-A1) and chloroquine (CQ) and the proteasome inhibitor MG132 were added to IPZ-treated cells. Both kinds of inhibitors further increased IPZ-induced protein ubiquitination, LC3B expression, viability inhibition, and apoptosis (Fig. 6e, f). These results suggest that autophagy-lysosome-mediated and proteasome-mediated degradation pathways are needed for the clearance of abnormal proteins and survival in KPNB1-deficient glioblastoma cells.

### ABT-263-potentiated apoptosis induced by KPNB1 depends on UPR

**Fig. 7:**
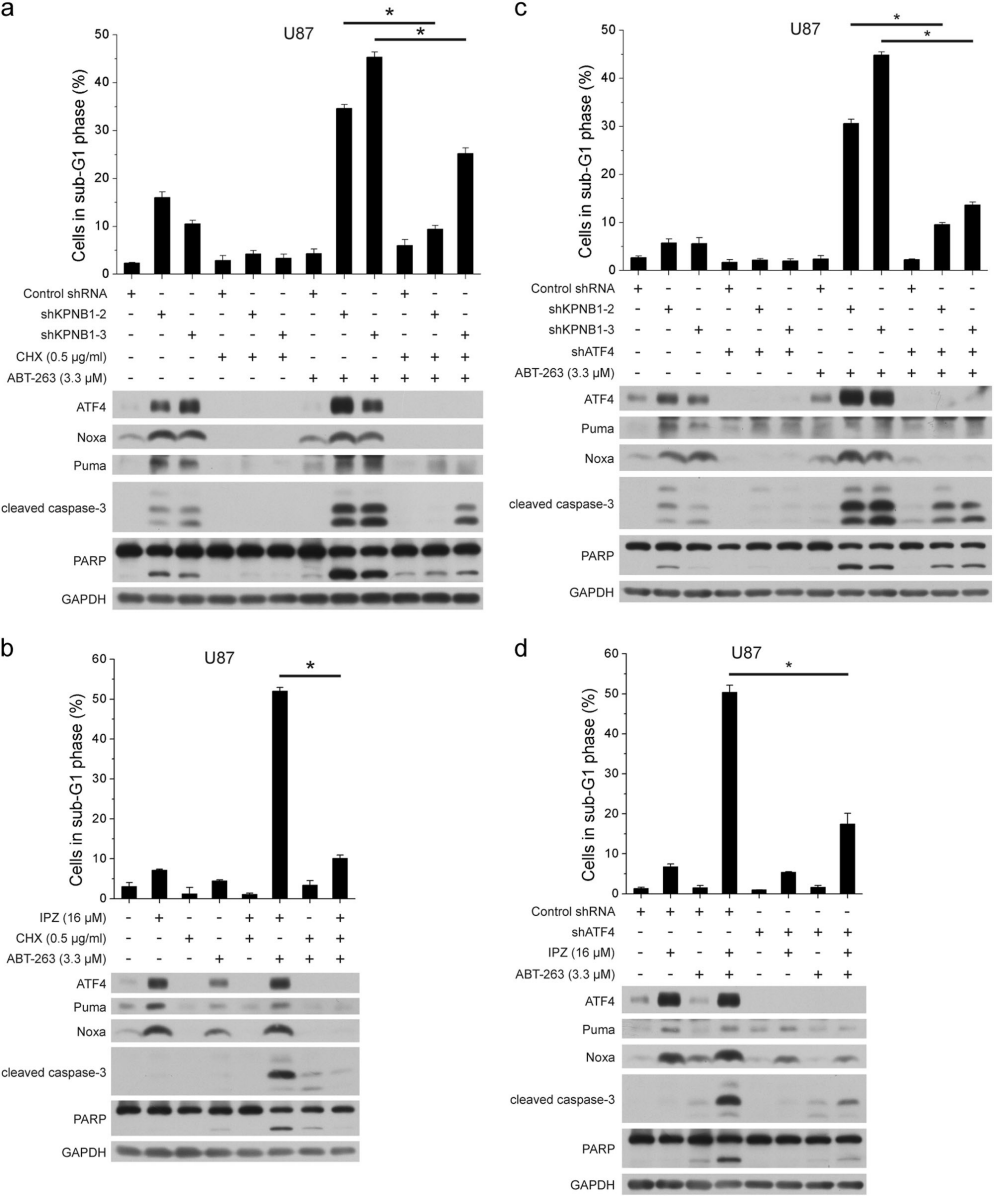
ABT-263-potentiated apoptosis induced by KPNB1 depends on UPR. **a** U87 cells expressing shKPNB1s were treated with CHX (0.5 μg/ml) and/or ABT-263 (3.3 μM) for 24 h. The sub-G1 population and protein levels were analyzed by flow cytometry (*n* = 2, mean ± s.d.) and western blots, respectively. **b** U87 cells were treated with IPZ (16 μM) and/or CHX (0.5 μg/ml) for 24 h and then treated with or without ABT-263 (3.3 μM) for another 24 h, before subjected to flow cytometry (*n* = 2, mean ± s.d.) and western blots. **c** U87 cells expressing shKPNB1s along with shATF4 were treated with or without ABT-263 (3.3 μM) for 24 h and subjected to flow cytometry (*n* = 2, mean ± s.d.) and western blots. **d** U87 cells expressing shATF4 were treated with or without IPZ (16 μM) for 24 h and then treated with or without ABT-263 (3.3 μM) for another 24 h, before subjected to flow cytometry (*n* = 2, mean ± s.d.) and western blots. For western blots, GAPDH was used as the loading control. *P < 0.05

To investigate whether UPR is essential for ABT-263 to enhance apoptosis induced by KPNB1 inhibition, low dose of CHX was added to shKPNB1 or IPZ-treated U87 cells culture in the presence of ABT-263. CHX treatment compromised ABT-263-induced apoptosis in shKPNB1 or IPZ-treated cells (Fig. 7a, b). Similarly, knockdown of ATF4 also disabled ABT-263 to trigger apoptosis in shKPNB1 or IPZ-treated U87 cells (Fig. 7c, d). In summary, our results suggest that the pro-apoptotic effect of KPNB1 inhibition and ABT-263 depends on UPR through the effect of ATF4.

## Discussion

**Fig. 8:**
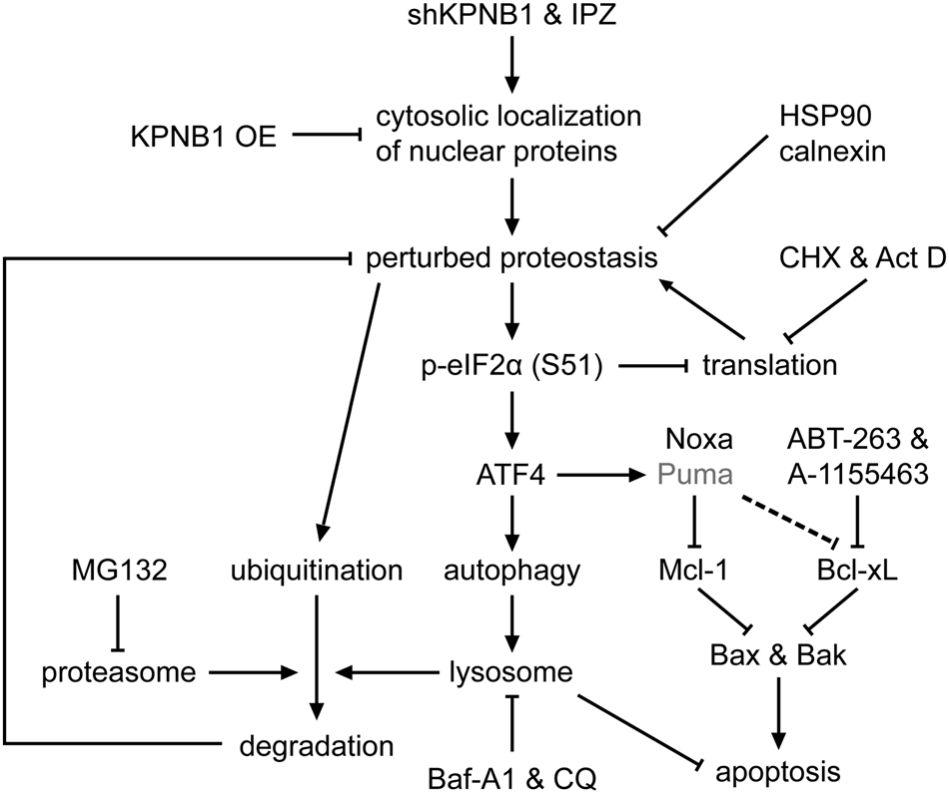
The mechanism of KPNB1 inhibition-induced apoptosis in glioblastoma cells. KPNB1 inhibition results in cytosolic localization of its cargos, which perturbs proteostasis and activates both UPR and protein degradation pathways to relieve protein overload. Blockade of protein degradation by inhibitors of proteasome or lysosome aggravates accumulation of ubiquitinated proteins and potentiates IPZ-induced apoptosis, whereas blockade of protein synthesis by inhibitors of translation or transcription has opposite effects. Failure of proteostasis recovery sustained activates eIF2α/ATF4 signaling, which upregulates BH3-only proteins and induces adaptive autophagy. Noxa and/or Puma compromise the binding of Mcl-1 and/or Bcl-xL to Bax and Bak, thus promoting apoptosis that can be enhanced by Bcl-xL inhibitors

Inhibition of KPNB1 has been implicated to selectively induce apoptosis in cancerous cells [22, 23, 26], but evidences for the underlying mechanism are rather inconclusive. So far, multiple studies suggest that KPNB1 inhibition-induced growth arrest and apoptosis rely on the reduction of nuclear import and activity of NF-κB p65 [23–25], while the other holds that mitotic arrest and decreased Mcl-1/Noxa ratio are the major reasons [22]. Our results in glioblastoma cells demonstrate that KPNB1 inhibition perturbs proteostasis, triggers the autophagy-lysosomal and ubiquitin-proteasomal-mediated protein degradation, and probably chaperone-mediated protein folding for adaption to stress. It also activates PERK branch of UPR that cause imbalance between anti-apoptotic and pro-apoptotic members of Bcl-2 protein family. Disability of Mcl-1 to sequester Bax and Bak is primarily responsible for apoptosis and vulnerability to Bcl-xL inhibitors (Fig. 8).

Importantly, inhibition of KPNB1 promotes glioblastoma cell apoptosis without evident prior mitotic arrest, which resembles observations in breast cancer cells but not in cervical cancer cells [22, 26], suggesting a cell-type-specific manner. In KPNB1-knockdown U87 cells, the dissipation of MAD2 and BubR1 occurs before the expression culmination of mitotic proteins at early times. However, in KPNB1-knockdown U251 cells or IPZ-treated U87 and U251 cells, mitotic proteins never accumulated but diminished progressively. The weak SAC activity may not protect mitotic proteins from APC/C^CDC20^ complex-mediated degradation before their levels reach thresholds causing robust mitotic arrest [35]. For this reason, downregulation of the short-lived protein Mcl-1 in KPNB1-deficient cells is unlikely resulted from prolonged mitotic arrest [22], but probably from UPR-induced translation arrest as described below. On the other hand, KPNB1 inhibition-induced UPR upregulates pro-apoptotic factors and p27 simultaneously [36], resulting in either G1 phase arrest for a period or not depending on the speed and intensity of UPR-induced apoptosis. Therefore, the increase of sub-G1 population accompanies the reduction of G1 phase population in both cell lines at later times.

Inhibition of the central regulator of protein nuclear import KPNB1 may cause cytosolic accumulation of proteins should have localized in the nucleus, resulting in cytosolic protein overload. This postulation is supported by the upregulated polyubiquitination of both p65 and total proteins, ALIS formation, and upregulated p65/chaperones interactions in KPNB1-deficient glioblastoma cells. Stresses like protein overload or misfolding induce transient aggregation of ubiquitinated proteins, namely ALIS, which relies on the lysosme for clearance [31]. ALIS may here serve as “overflow stations” to make room for excessive cytosolic proteins. Meanwhile, protein overload may be buffered by chaperones like HSP90 and calnexin abundant in the cytosol and ER, respectively, which protects excessive KPNB1 cargos from ubiquitin-proteasomal degradation and survival [37, 38]. Protein overload by, i.e., MG132, also disturbs proteostasis in the ER, which activates UPR to halt protein translation and alleviates ER stress [39]. As a stress transducer of UPR, PERK ameliorates ER protein overload by inhibiting phosphorylation of the translation initiation factor eIF2α. Phosphorylated eIF2α enhances ATF4 translation, which in turn activates the transcription of CHOP, ATF3, GADD34, and BH3-only proteins Puma and Noxa to govern apoptosis [32, 40]. We found that KPNB1 inhibition activated PERK/eIF2α/ATF4 signaling in both human and rat glioblastoma cells. Attenuation of protein synthesis by CHX or Act D reversed polyubiquitinated protein accumulation, PERK branch activation, and apoptosis in KPNB1-deficient cells, which resembled observations in MG132-treated cells [39], suggesting that KPNB1 inhibition-induced UPR is resulted from protein overload. In consistent with the previous study [41], KPNB1 inhibition-induced upregulation of Noxa and Puma and apoptosis in glioblastoma cells depends on ATF4 but not CHOP. This is because nuclear import of CHOP is mediated by KPNB1. Intriguingly, KPNB1 inhibition-induced upregulation of ATF3 but not CHOP was mediated by ATF4. Therefore, CHOP upregulation might be mediated by other factors like XBP1s [42]. To restore proteostasis, autophagy-lysosomal and proteasomal pathways were activated for protein degradation, of which the former is regulated by ATF4 [33, 34]. Blockade of protein degradation by inhibitors of these two pathways potentiated IPZ-induced apoptosis, suggesting these pathways cause IPZ resistance. Given that existing KPNB1 inhibitors are not that efficacious, combining lysosome or proteasome inhibitors may improve the efficacy and selectivity of KPNB1 inhibitors in cancer treatment [43].

UPR-induced upregulation of Puma, Noxa, and Bak results in the remodeling of mitochondrial network in KPNB1-deficient glioblastoma cells. Noxa preferentially binds Mcl-1 and sometimes dissociates from Mcl-1 to bind and inhibit Bcl-xL [44]. Puma binds Bcl-2, Bcl-xL, and Mcl-1. These bindings release sequestered Bax and Bak. Freed Bax and Bak then undergo conformational changes, homodimerization, and heterodimerization to become activated, thus eliciting MOMP [29]. Moreover, Noxa, Puma, and Bim can directly bind and activate Bax and Bak [45, 46]. Noxa upregulation and disrupted bindings of Mcl-1 to Bax and Bak contribute to both KPNB1 knockdown and IPZ-induced apoptosis, whereas Puma upregulation and disrupted bindings of Bcl-xL to Bax or Bak simply contribute to KPNB1 knockdown-induced apoptosis. One explanation is that the tolerances for different BH3-only proteins vary among different cell types [47]. U87 and U251 cells may tolerate higher levels of Puma than Noxa. KPNB1 knockdown partly inactivates Mcl-1 and Bcl-xL, respectively, by Mcl-1 downregulation and p-Bcl-xL (S62) and upregulates Bak, lowering the tolerance of cells to the upregulation of Noxa and Puma. IPZ treatment exerts less effect on the expression of Mcl-1 and Bak and phosphorylation of Bcl-xL (data not shown), making BH3-only protein tolerance largely unchanged. In this case, Noxa/Mcl-1 interaction but not bindings of Puma to Mcl-1 and Bcl-xL confer to IPZ-induced apoptosis. It is noteworthy that Noxa/Mcl-1 in shKPNB1 U87 cells was not increased. As a consequence, shNoxa reversed the apoptosis in these cells less efficaciously than shPuma.

KPNB1 inhibition abrogates ABT-263 resistance in glioblastoma cells. ABT-263 is an orally available Bad-like BH3 mimetic that binds and neutralizes Bcl-2 and Bcl-xL but not Mcl-1 [29]. Although ABT-263 shows activity against lymphoid malignancies in clinical trials, its application is limited because high-dose treatment of ABT-263 induces thrombocytopenia, which is due to the critical role of Bcl-xL in platelet survival [48]. ABT-199, a Bcl-2 selective inhibitor, does not induce thrombocytopenia and is clinically applied to treat chronic lymphocytic leukemia patients [29]. However, ABT-199 shows limited efficacy in most cancers due to the functional redundancy of Bcl-2, Bcl-xL, and Mcl-1 in anti-apoptosis. Moreover, ABT-199 induces neutropenia in solid tumors [48, 49]. Combination regimens that can reduce the effective dosage of ABT-263 to the therapeutic window for Bcl-xL inhibition will contribute to overcome resistance and avoid adverse effects. Mcl-1 is a major determinant for ABT-263 response. Inactivation of Mcl-1 either by decreasing its expression or binding to pro-apoptotic proteins restores ABT-263 sensitivity in cancer cells [50–52]. In glioblastoma cells, KPNB1 depletion inactivates Mcl-1 by downregulating Mcl-1 expression and inducing expression of Mcl-1-bound BH3-only proteins, leading to the activation of Bax and Bak. Bcl-xL is more important than Bcl-2 for the survival of solid tumors [53]. Inhibitors of Bcl-xL rather than Bcl-2 enhances KPNB1 inhibitor-induced apoptosis in glioblastoma cells, suggesting that combination of Bcl-xL inhibitors and KPNB1 inhibitors, such as IPZ and INI-43 [43], may be efficacious and safe for solid tumors therapy.

In summary, our studies identify KPNB1 as a target for apoptosis induction in glioblastoma cells and suggest that KPNB1 inhibition, alone or in combination with inhibitors of Bcl-xL, lysosome, or proteasome, may serve as promising therapeutic strategies for glioblastoma treatment.

## Materials and methods

### Cell culture

Human glioblastoma cell lines U87 and U251 were cultured in DMEM supplemented with 10% fetal bovine serum (FBS), 1% non-essential amino acid, and 1% sodium pyruvate (Life Technologies, Grand Island, USA). Human glioblastoma cell lines A172 and SHG-44 were cultured in DMEM with 10% FBS. Human fetal astrocytes from cerebral cortex were cultured in astrocyte medium (ScienCell Research Laboratories, Carlsbad, USA) with 2% FBS. Rat glioblastoma cell line C6 and rat primary glia were cultured in DMEM/F12 (Life Technologies) with 10% FBS. Cell lines were obtained from Shanghai Institute of Biochemistry and Cell Biology (Shanghai, China) and authenticated by STR profiling. Human astrocytes were obtained from ScienCell Research Laboratories. Cells were tested for mycoplasma contamination. All cells were maintained under standard cell culture conditions at 37 °C and 5% CO_2_.

### Antibodies and reagents

Primary antibodies used in this study were listed below: antibodies against α-tubulin (HRP-conjugated) (HRP-66031), BubR1 (11504-2-AP), CD133 (18470-1-AP), GFAP (6190-1-Ig), KPNB1 (10077-1-AP), MAD2 (10337-1-AP), Tuj1 (10094-1-AP) (ProteinTech Group, Wuhan, China), p-IRE1α (S724) (ab124945), ubiquitin (Alexa Fluor 647) (ab205468) (Abcam, Canbridge, UK), ATF4 (11815), ATF6 (65880), Atg3 (3415), Atg5 (12994), Atg7 (8558), Bax (5023), Bak (12105), Bcl-2 (4223), Bcl-xL (2764), Bim (2933), calnexin (2679), caspase-3 (9662), CHIP (2080), CHOP (2895), Histone H3 (3377), HSC70/HSPA8 (8444), HSP70 (4872), HSP90 (4877), LC3B (3868), Mcl-1 (5453), MeCP2 (3456), Noxa (14766), Oct4 (2750), p62 (8025), p62 (Alexa Fluor 488) (8833), NF-kB p65 (8242), PARP (9532), p-Aurora A (T288)/Aurora B (T232)/Aurora C (T198) (2914), p-Histone H3 (S10) (4499), p-PERK (T980) (3179), Puma (12450), ubiquitin (HRP-conjugated) (14049), XBP1s (12782) (Cell Signaling Technology, Beverly, USA), Bax (6A7) (556467), Mcl-1 (for immunoprecipitation) (559027) (BD Biosciences, San Jose, USA), Bak, NT (06-536) (Merck Millipore, Darmstadt, Germany), ATF3 (BS2261), cyclin B1 (BS1392), eIF2α (BS3651), p27 (BS3714), p-Bcl-xL (S62) (BS4025), p-eIF2α (S51) (BS4787), PERK (BS2156) (Bioworld Technology, Nanjing, China), CDC2 (AF0778), p-CDC2 (T161) (AF8001) (Affinity Biologicals, Zhenjiang City, China), and GAPDH (KC-5G5) (Kangchen, Shanghai, China). Anti-mouse (7076) or anti-rabbit (7074) secondary antibodies (horseradish peroxidase-conjugated) were acquired from Cell Signaling Technology. Alexa Fluor 546 goat anti-rabbit antibody (A-11010) was from Life Technologies (Carlsbad, USA).

Reagents and kits used in this study were listed below: IPZ (Merck Millipore), propidium iodide, thymidine (Sigma-Aldrich, St. Louis, USA), protease inhibitor cocktail, RNase A (Thermo Scientific, Waltham, USA), phosphatase inhibitor, thiazolyl blue tetrazolium bromide (Sangon, Shanghai, China), cell lysis buffer for Western and IP, CHX, cytosolic and mitochondrial protein extraction kit, Hoechst 33342, mitochondrial membrane potential assay kit with JC-1, nuclear and cytoplasmic protein extraction kit, PMSF, RIPA (Beyotime, Nantong, China), protein A agarose, protein G agarose (Roche Diagnositics, Indianapolis, USA), ABT-199, ABT-263, Baf-A1, MG132, nocodazole (Selleck, Shanghai, China), 17-AAG, A-1155463, Act D, CQ diphosphate (Medchem Express, Monmouth Junction, USA), INI-43 (3-(1H-benzimidazol-2-yl)-1-(3-dimethylaminopropyl)pyrrolo[5,4-b]quinoxalin-2-amine) (ZINC identification no. 20547783) (InterBioScreen, Russia).

### Generation of gene-specific lentiviruses

shRNAs targeting human KPNB1, Bax, Bak, Bcl-xL, Mcl-1, ATF4, CHOP, and ATF3, and a scrambled (control) shRNA were cloned into the lentiviral vector pLKD-CMV-GFP-U6-shRNA. Human 3Flag-tagged KPNB1 gene, untagged phospho-defective Bcl-xL (S62A) mutant and non-degradable phospho-defective Mcl-1 (T92A) mutant were generated by PCR amplification. KPNB1 and Bcl-xL (S62A) were cloned into the lentiviral vector pLOV-CMV-eGFP and Mcl-1 (T92A) was cloned into pLOV-EF1a-eGFP. Lentiviruses encoding various shRNA plasmids and expression plasmids were generated as previous described [54]. The sequence of shRNAs used were listed in the Supplementary Information.

### Western blots

After collection, cells were lysed in RIPA buffer supplemented with PMSF, phosphatase inhibitor, and protease inhibitor cocktail. Western blots were carried out in compliance with the standard protocol.

### MTT assays and colony formation assays

MTT assays and colony formation assays were performed as previously described [54]. Three independent assays were repeated.

### Flow cytometry

Cell cycle and mitochondrial membrane potential analysis were carried out using LSR-II. Cell cycle distribution was determined as previously described [54]. Mitochondrial membrane potential was determined using mitochondrial membrane potential assay kit with JC-1 according to the manufacturer’s protocol. Data were analyzed by FlowJo software (Ashland, USA). Three independent assays were repeated.

### Immunoprecipitation

Cells were washed by phosphate-buffered saline (PBS) and lysed on ice using cell lysis buffer for Western and IP (to detect interacting proteins) or RIPA (to detect p65 ubiquitination). Lysates were adjusted to equal protein concentrations and incubated with indicated antibodies at 4 °C overnight, followed by incubation with protein A or protein G agarose at 4 °C for 2 h. Precipitates were then washed four times with cell lysis buffer and analyzed by standard Western blots.

### Immunofluorescence

Cells plated onto glass coverslips were fixed with 4% paraformaldehyde. Cells were incubated with blocking buffer (3% bovine serum albumin and 0.1% Triton in PBS) for 1 h and stained with antibodies and Hoechst 33342 overnight. Fluorescent signals were observed with a Nikon FN1 confocal microscope at ×60 magnification.

### Statistical analysis

GraphPad Prism 6.01 software (GraphPad Software, Inc., San Diego, USA) was used in the study. The two-tailed unpaired *t*-test was used to determine significant differences between the mean values of groups, with statistical significance defined as *P* < 0.05. The variance is similar between groups that are being statistically compared. Sample size in the cell cycle analysis is calculated by Sample Size Calculator (http://www.calculator.net/sample-size-calculator.html).

## Electronic supplementary material

supplementary information

supplementary Figure 1-4

supplementary Figure 5

Full blots for western blot
