# Supplementary material for: KPNB1 inhibition disrupts proteostasis and triggers unfolded protein response-mediated apoptosis in glioblastoma cells
Source: Oncogene. 2018 Mar 9;37(22):2936–52. doi: 10.1038/s41388-018-0180-9 (PMC5978811; doi:10.1038/s41388-018-0180-9)
Supplement: Supplementary file 1 — supplementary information [file 41388_2018_180_MOESM1_ESM.doc]

**Supplementary Information**

The sequence of shRNAs used were listed as follows: shKPNB1-1, CCGGGCTTGCTATTGATGCTAATGCCTCGAGGCATTAGCATCAATAGCAAGCTTTTTTG; shKPNB1-2, CCGGGGAAGTGTTGGGTGGTGAATTCTCGAGAATTCACCACCCAACACTTCCTTTTTTG; shKPNB1-3, CCGGGGAAGTGTTGGGTGGTGAATTCTCGAGAATTCACCACCCAACACTTCCTTTTTTG; shBax, CCGGGCTCTGAGCAGATCATGAATTCAAGAGATTCATGATCTGCTCAGAGCTTTTTTG; shBak, CCGGCCCATTCACTACAGGTGAATTCAAGAGATTCACCTGTAGTGAATGGGTTTTTTG; shBcl-xL, CCGGCTGCTTGGGATAAAGATGCAACTCGAGTTGCATCTTTATCCCAAGCAGTTTTTTG; shMcl-1, CCGGCCCTAGCAACCTAGCCAGAAACTCGAGTTTCTGGCTAGGTTGCTAGGGTTTTTTG; shATF4, CCGGGGATAGTCAGGAGCGTCAATGCTCGAGCATTGACGCTCCTGACTATCCTTTTTTG; shCHOP, CCGGGCCAATGATGTGACCCTCAATCTCGAGATTGAGGGTCACATCATTGGCTTTTTTG; shATF3, CCGGGCACCTCTGCCACCGGATGTTCAAGAGACATCCGGTGGCAGAGGTGCTTTTTTG; shNoxa, CCGGGCAAGAACGCTCAACCGAGTTCAAGAGACTCGGTTGAGCGTTCTTGCTTTTTTG; shPuma, CCGGGGGACTTTCTCTGCACCATGTCTCGAGACATGGTGCAGAGAAAGTCCCTTTTTTG; scrambled shRNA, CCGGTTCTCCGAACGTGTCACGTTTCAAGAGAACGTGACACGTTCGGAGAATTTTTTG.

**Supplementary Figure Legends**

**Supplementary Figure 1** *KPNB1* mRNA expression correlates with glioma progression and predicts poor survival. (**a**) *KPNB1* mRNA expression in GBM, oligodendroglioma, astrocytoma and normal brain, as reported by the REMBRANDT knowledgebase. Each dot represents a single sample and the line represents the mean value for each category. (**b**) Kaplan-Meier plots of correlations between *KPNB1* expression and survival probability of glioma patients from all glioma samples (upper panel) and GBM subsets (lower panel).

**Supplementary Figure 2** Western blots of HA, U87 and U251 cells for their glial stemness. GAPDH was used as the loading control.

**Supplementary Figure 3** Mitochondrial membrane potential analysis of shKPNB1-expressing or IPZ (16 μM)-treated U87 and U251 cells.

**Supplementary Figure 4** U87 and U251 cells expressing shKPNB1s were infected by lentivirus encoding shBcl-xL or shMcl-1, followed by western blots. GAPDH was used as the loading control.

**Supplementary Figure 5** (**a**) Western blots analysis of cytosol and nucleus p65 level in shKPNB1-expressing U87 cells. (**b**) p65 was immunoprecipitated from shKPNB1-expressing U87 and U251 cells. Ubiquitin was identified by western blots. (**c**) U87 and U251 cells infected with lentiviruses encoding shKPNB1s were harvested at indicated time points before western blots. (**d**) U87 and U251 were co-treated with IPZ and 17-AAG at indicated concentration for 48h, followed by MTT assays (n=3, mean ± s.d.). (**e**) Rat astrocytes and C6 cells were treated with IPZ at indicated concentrations for 48 h, followed by MTT assays (n=3, mean ± s.d.). (**f**) U87 and U251 cells expressing shKPNB1s were treated with Act D (0.5 μg/ml) or CHX (U87: 0.5 μg/ml and U251: 2.5 μg/ml) and subjected to western blots. (**g**) U87 and U251 cells expressing shKPNB1s were treated with or without CHX and subjected to flow cytometry (n=2, mean ± s.d., *P<0.05, compared with respective shKPNB1 group) and western blots. (**h**) Western blots analysis of cytosolic and nuclear levels of ATF4, CHOP and ATF3 in shKPNB1-expressing U87 cells. (**i**) U87 and U251 cells expressing shKPNB1s along with shATF4, shCHOP or shATF3 were subjected to flow cytometry (n=2, mean ± s.d., *P<0.05, compared with respective shKPNB1 group) and western blots. For western blots, α-tubulin, MeCP2 and GAPDH were used as the loading control of cytosolic fraction, mitochondrial fraction and total cell lysate, respectively.
