# Supplementary figures and images for: KPNB1 inhibition disrupts proteostasis and triggers unfolded protein response-mediated apoptosis in glioblastoma cells
Source: Oncogene. 2018 Mar 9;37(22):2936–52. doi: 10.1038/s41388-018-0180-9 (PMC5978811; doi:10.1038/s41388-018-0180-9)

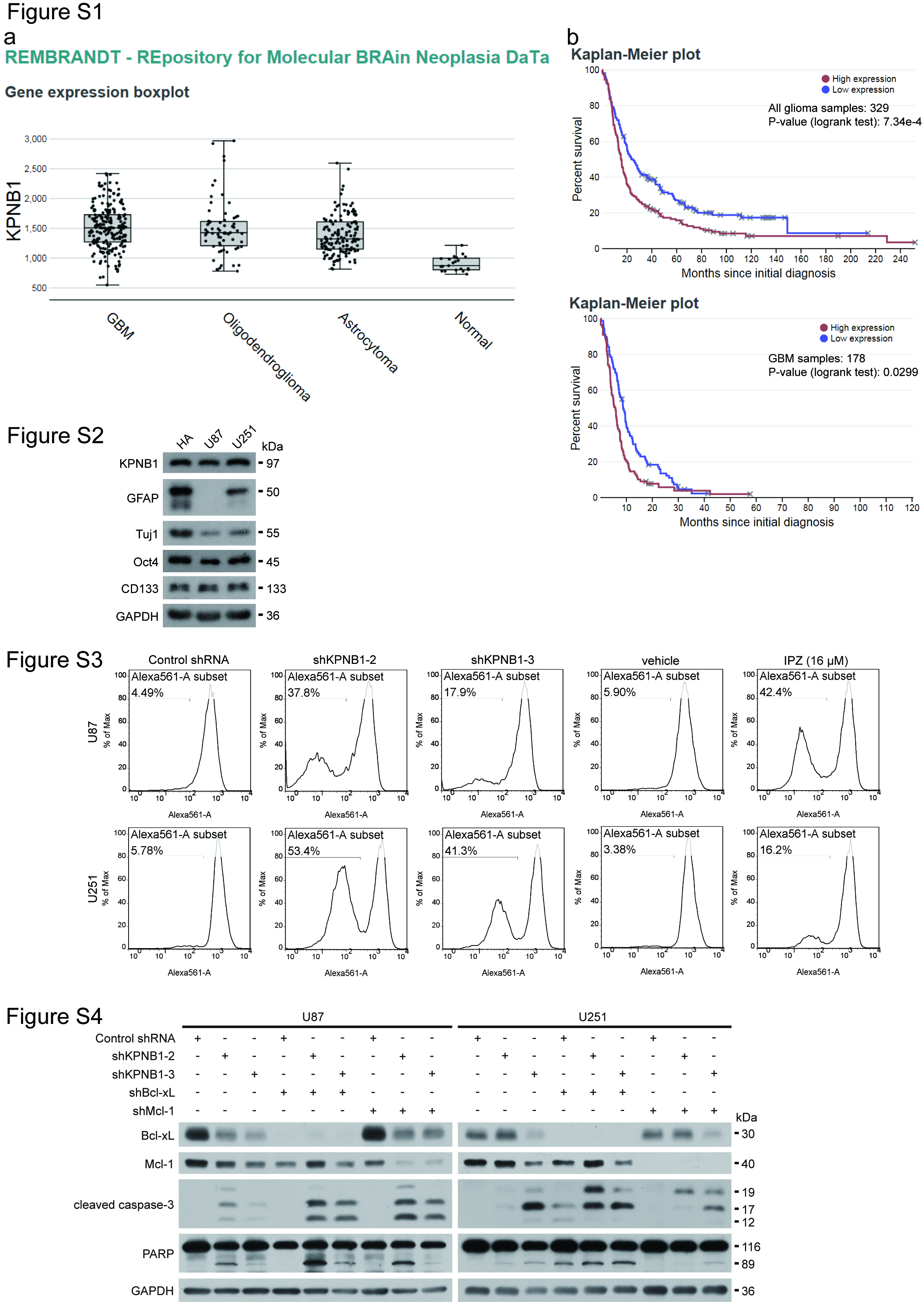

Supplement: Supplementary file 2 — supplementary Figure 1-4 [file 41388_2018_180_MOESM2_ESM.tif]

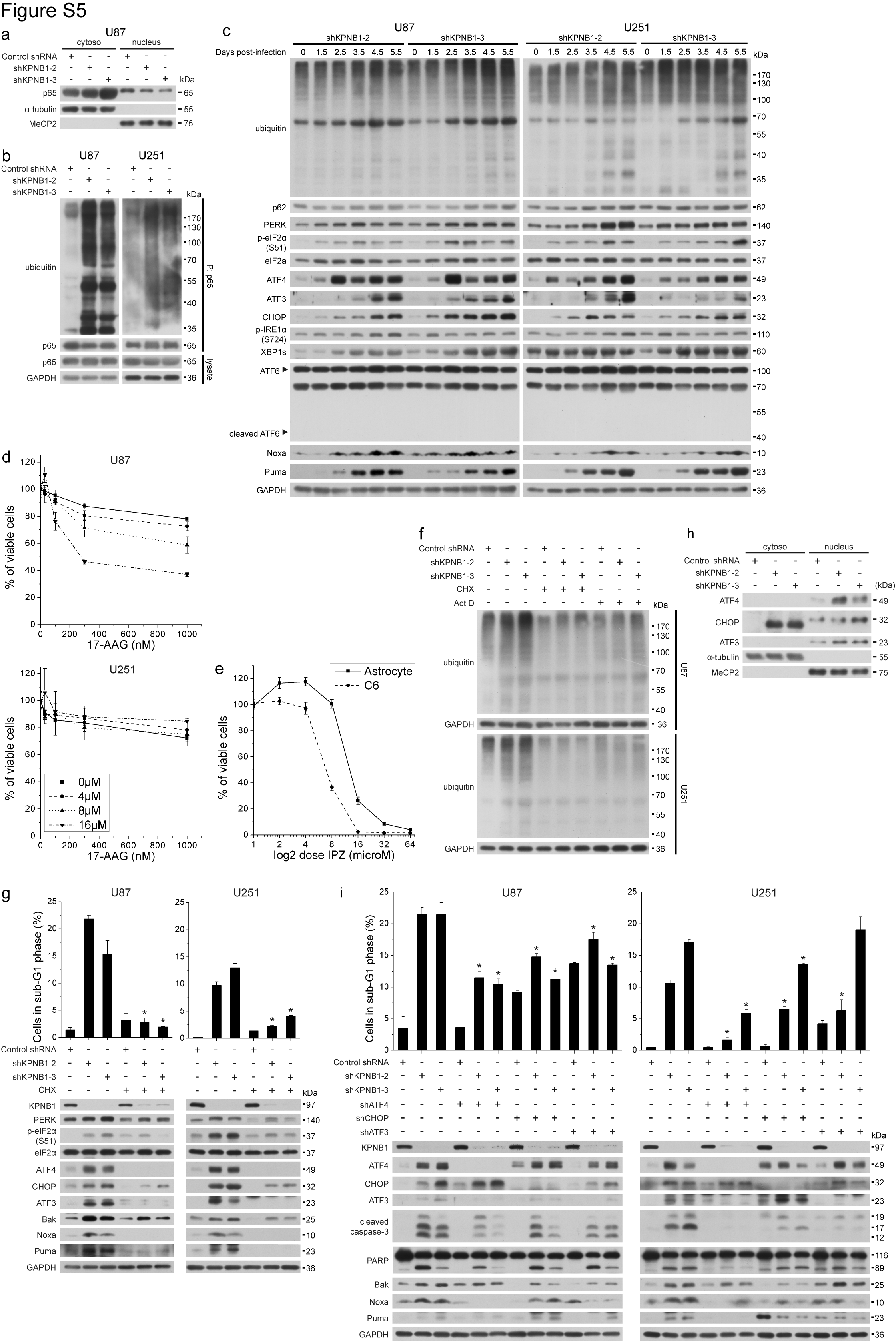

Supplement: Supplementary file 3 — supplementary Figure 5 [file 41388_2018_180_MOESM3_ESM.tif]
