# Supplementary material for: KPNB1 inhibition disrupts proteostasis and triggers unfolded protein response-mediated apoptosis in glioblastoma cells
Source: Oncogene. 2018 Mar 9;37(22):2936–52. doi: 10.1038/s41388-018-0180-9 (PMC5978811; doi:10.1038/s41388-018-0180-9)
Supplement: Supplementary file 4 — Full blots for western blot [file 41388_2018_180_MOESM4_ESM.pdf]

Figure 1a

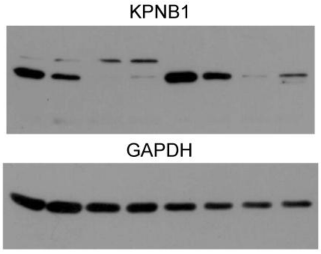

Figure 2e

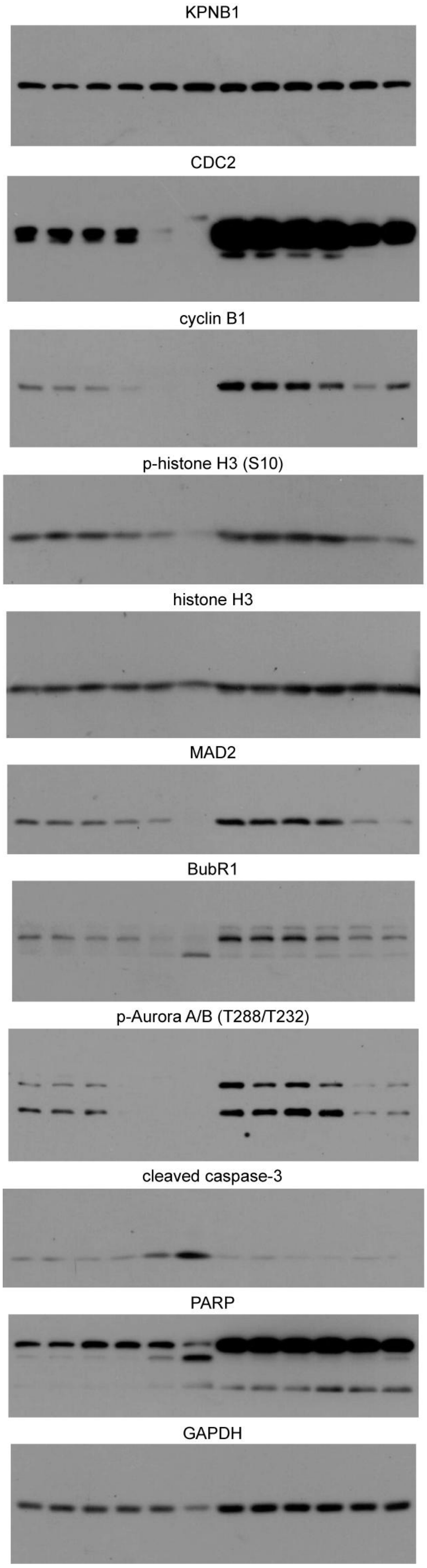

Figure 2b

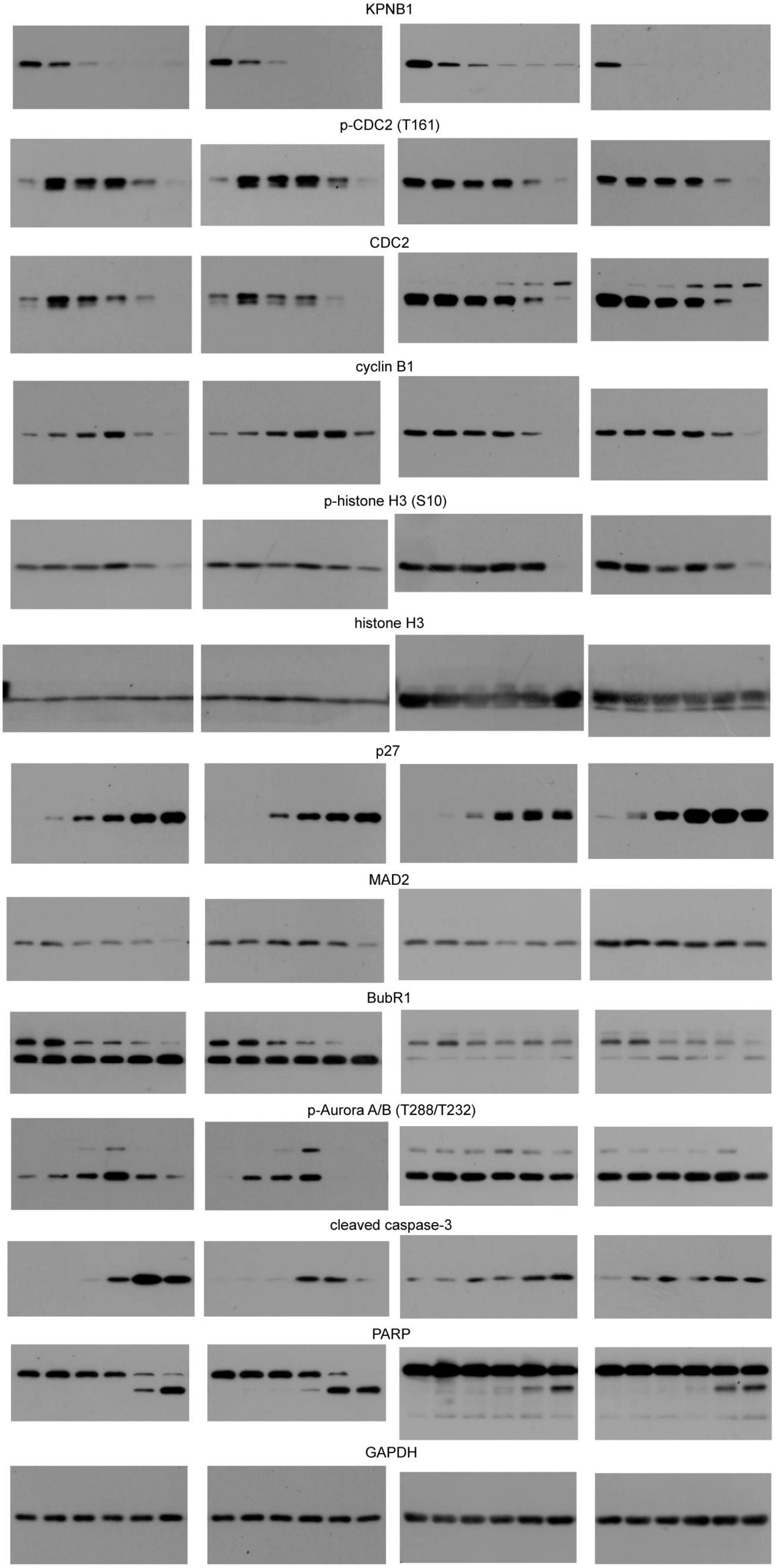

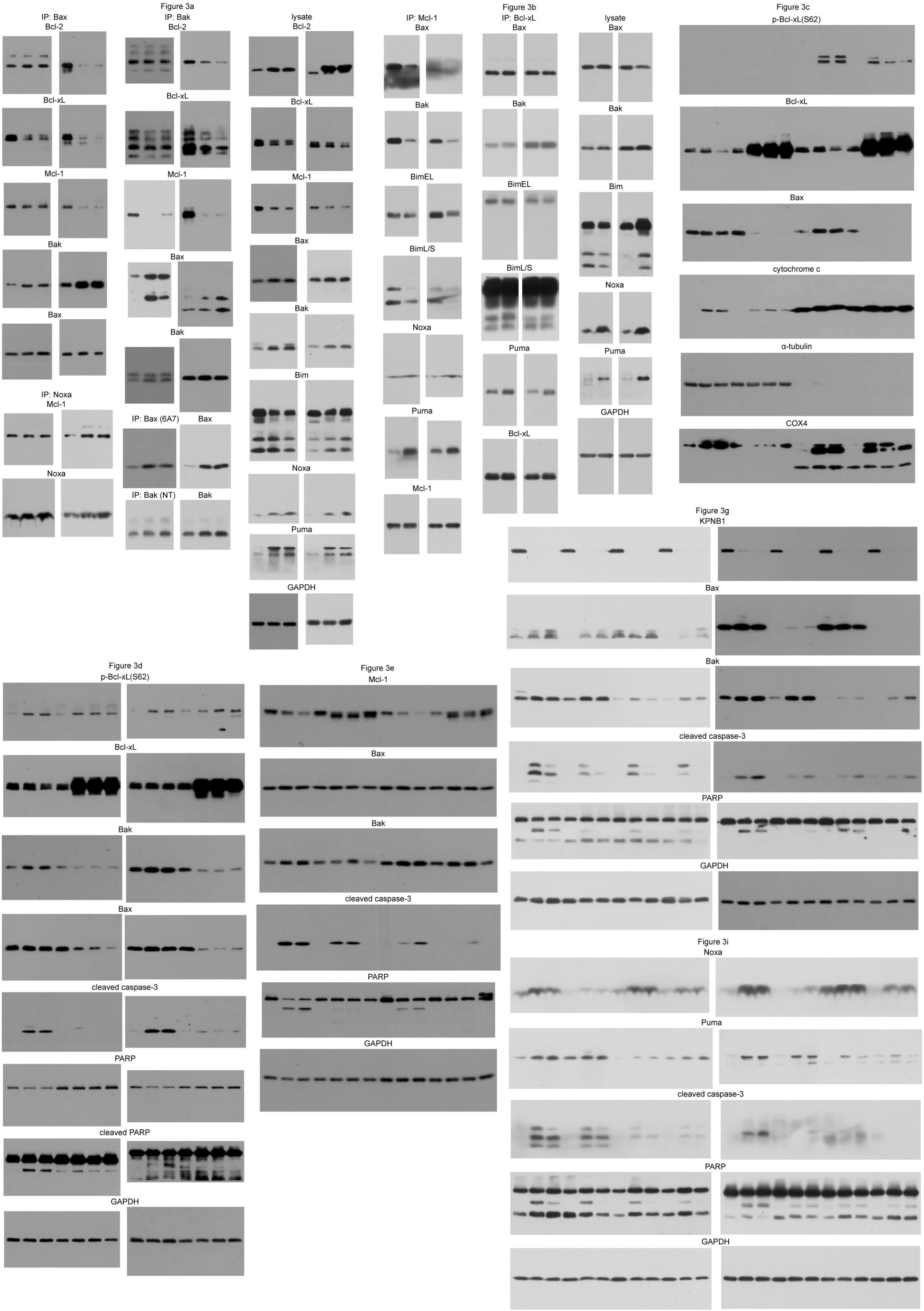
Figure 3a
IP: Bax
Bcl-2
Bcl-xL
Mcl-1
Bak
Bax
IP: Noxa
Mcl-1
Noxa
IP: Bak
Bcl-2
Bcl-xL
Mcl-1
Bax
Bak
IP: Bax (6A7)
Bax
IP: Bak (NT)
Bak
lysate
Bcl-2
Bcl-xL
Mcl-1
Bax
Bak
Bim
Noxa
Puma
GAPDH
Figure 3b
IP: Mcl-1
Bax
Bak
BimEL
BimL/S
Noxa
Puma
Mcl-1
IP: Bcl-xL
Bax
Bak
BimEL
BimL/S
Puma
Bcl-xL
lysate
Bax
Bak
Bim
Noxa
Puma
GAPDH
Figure 3c
p-Bcl-xL(S62)
Bcl-xL
Bax
cytochrome c
α-tubulin
COX4
Figure 3g
KPNB1
Bax
Bak
cleaved caspase-3
PARP
GAPDH
Figure 3d
p-Bcl-xL(S62)
Bcl-xL
Bak
Bax
cleaved caspase-3
PARP
cleaved PARP
GAPDH
Figure 3e
Mcl-1
Bax
Bak
cleaved caspase-3
PARP
GAPDH
Figure 3i
Noxa
Puma
cleaved caspase-3
PARP
GAPDH

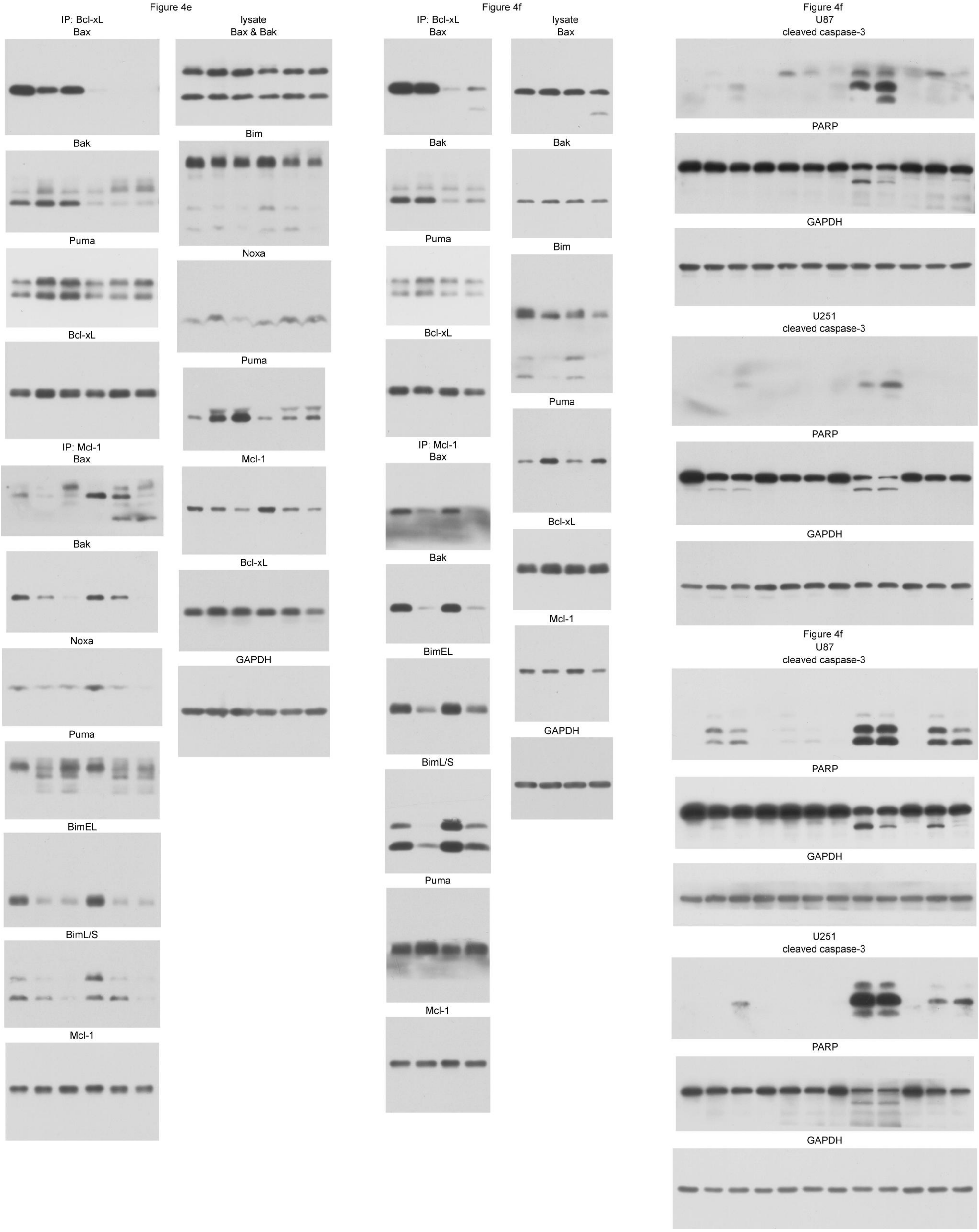

Figure 4e
IP: Bcl-xL
Bax
Bak
Puma
Bcl-xL
IP: Mcl-1
Bax
Bak
Noxa
Puma
BimEL
BimL/S
Mcl-1
lysate
Bax & Bak
Bim
Noxa
Puma
Mcl-1
Bcl-xL
GAPDH
Figure 4f
IP: Bcl-xL
Bax
Bak
Puma
Bcl-xL
IP: Mcl-1
Bax
Bak
BimEL
BimL/S
Puma
Mcl-1
lysate
Bax
Bak
Bim
Puma
Bcl-xL
Mcl-1
GAPDH
Figure 4f
U87
cleaved caspase-3
PARP
GAPDH
U251
cleaved caspase-3
PARP
GAPDH
Figure 4f
U87
cleaved caspase-3
PARP
GAPDH
U251
cleaved caspase-3
PARP
GAPDH

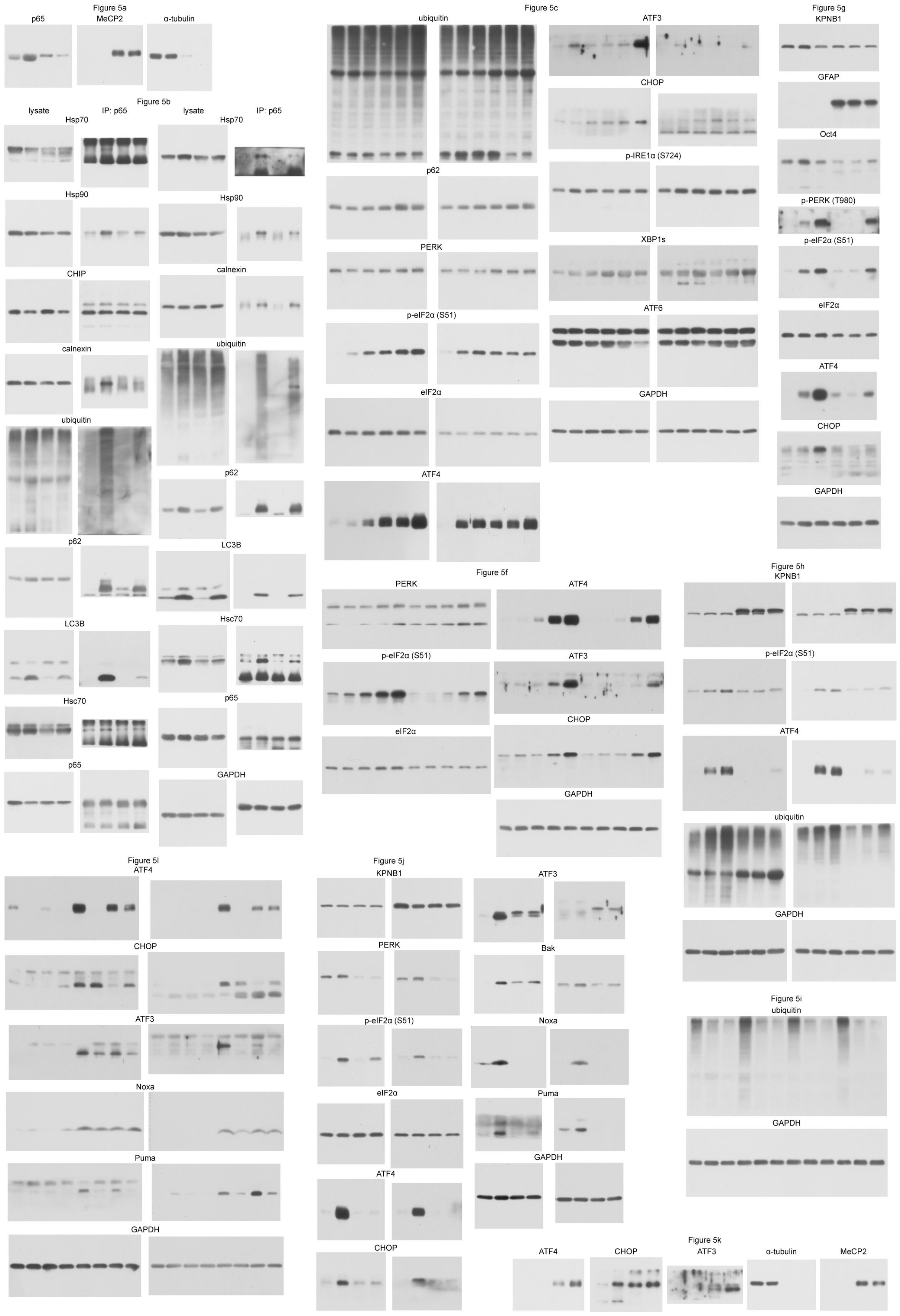
Figure 5a
p65
MeCP2
α-tubulin
Figure 5b
lysate
IP: p65
lysate
IP: p65
Hsp70
Hsp70
Hsp90
Hsp90
CHIP
calnexin
calnexin
ubiquitin
ubiquitin
p62
p62
LC3B
LC3B
Hsc70
Hsc70
p65
p65
GAPDH
Figure 5c
ubiquitin
ATF3
CHOP
p62
p-IRE1α (S724)
PERK
XBP1s
p-eIF2α (S51)
ATF6
eIF2α
GAPDH
ATF4
Figure 5g
KPNB1
GFAP
Oct4
p-PERK (T980)
p-eIF2α (S51)
eIF2α
ATF4
CHOP
GAPDH
Figure 5f
PERK
ATF4
p-eIF2α (S51)
ATF3
eIF2α
CHOP
GAPDH
Figure 5h
KPNB1
p-eIF2α (S51)
ATF4
ubiquitin
GAPDH
Figure 5l
ATF4
CHOP
ATF3
Noxa
Puma
GAPDH
Figure 5j
KPNB1
ATF3
PERK
Bak
p-eIF2α (S51)
Noxa
eIF2α
Puma
GAPDH
ATF4
CHOP
Figure 5i
ubiquitin
GAPDH
Figure 5k
ATF4
CHOP
ATF3
α-tubulin
MeCP2

Figure 6a

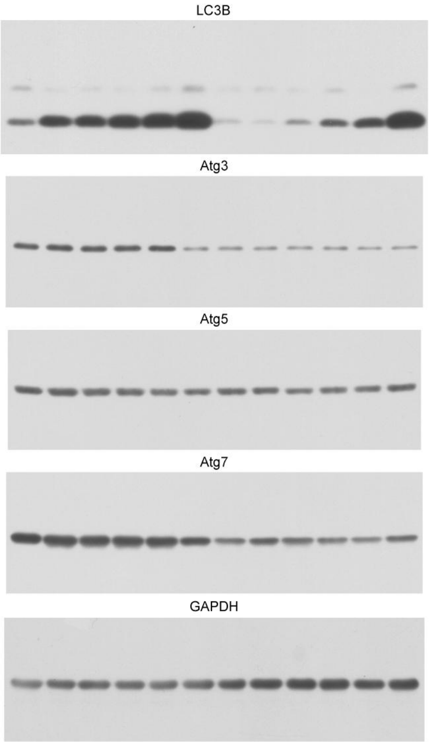

Figure 6b

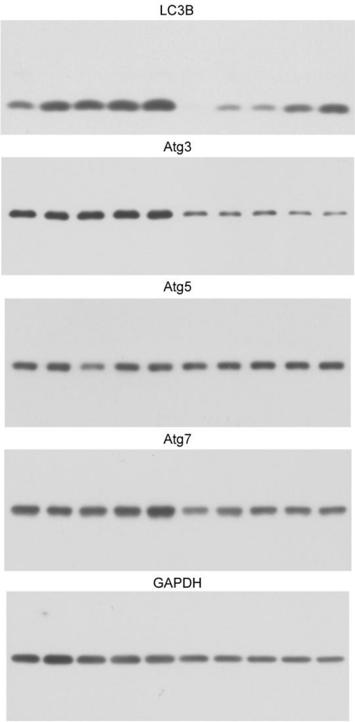

Figure 6c

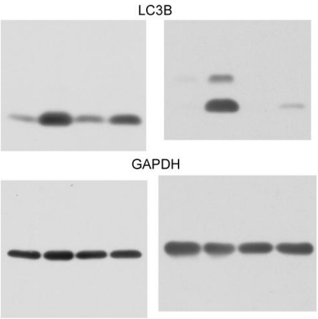

Figure 6c

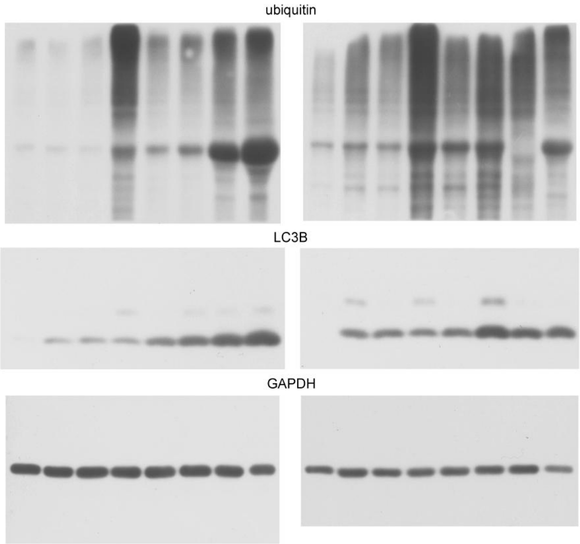

Figure 6c

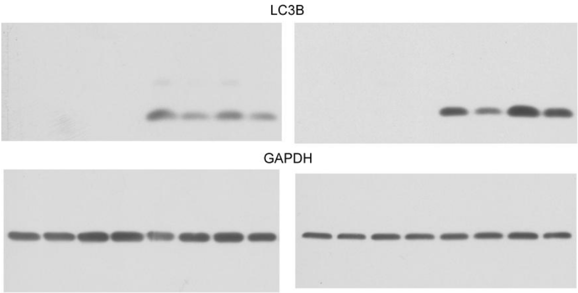

Figure 7a

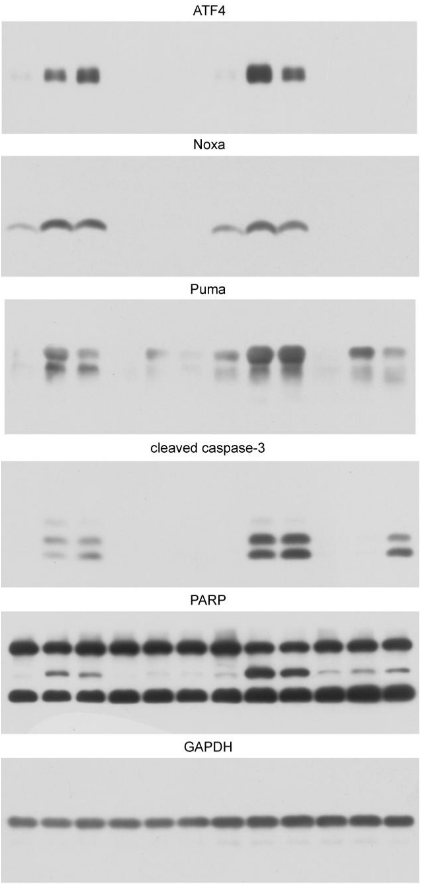

Figure 7b

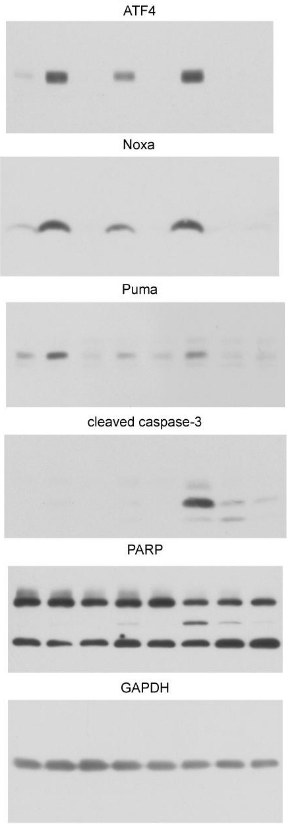

Figure 7c

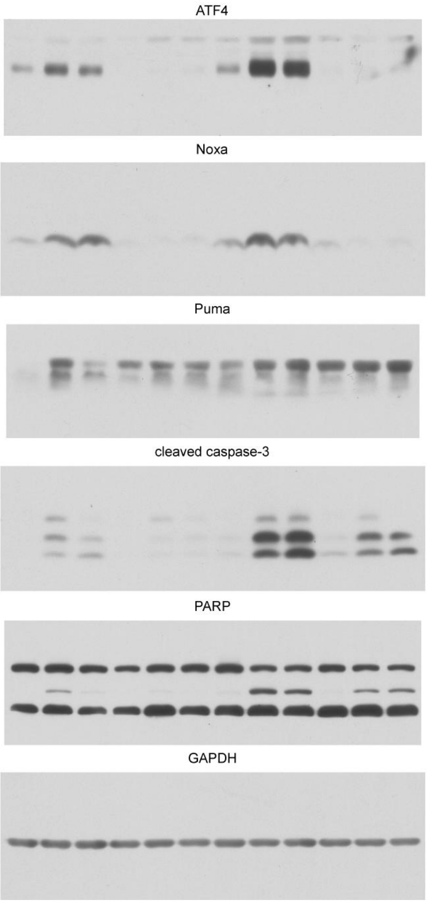

Figure 7d

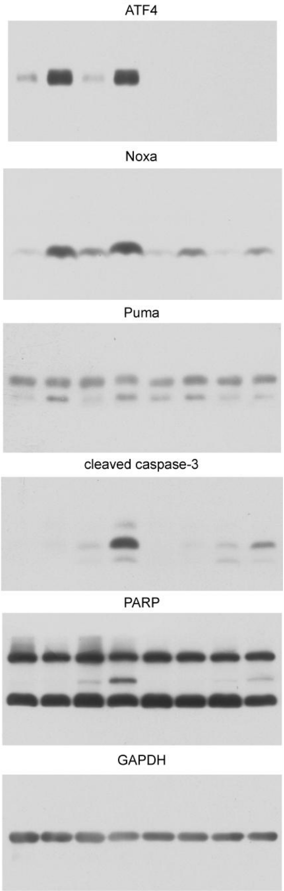

Figure S2

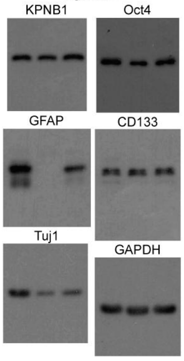

Figure S4

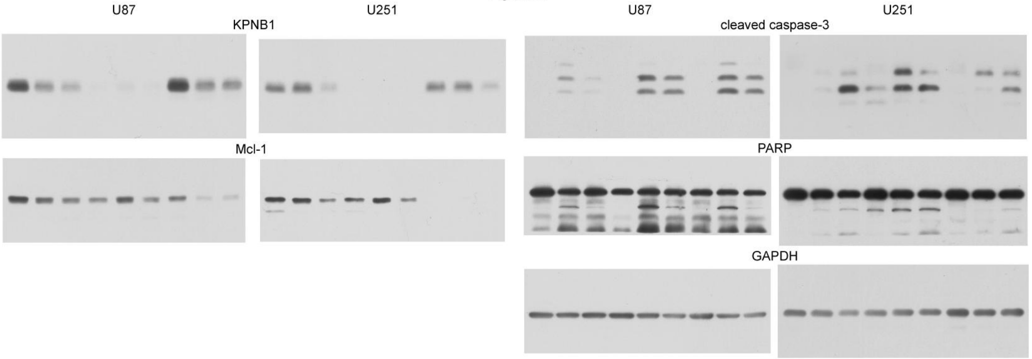

Figure S5a

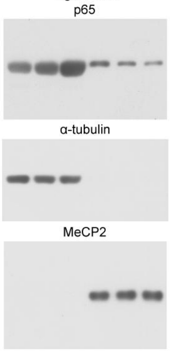

Figure S5b

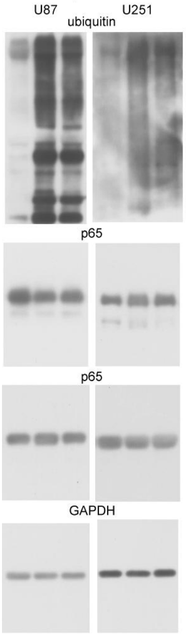

Figure S5h

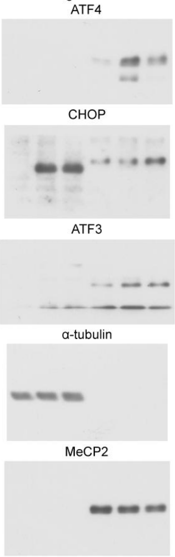

Figure S5c

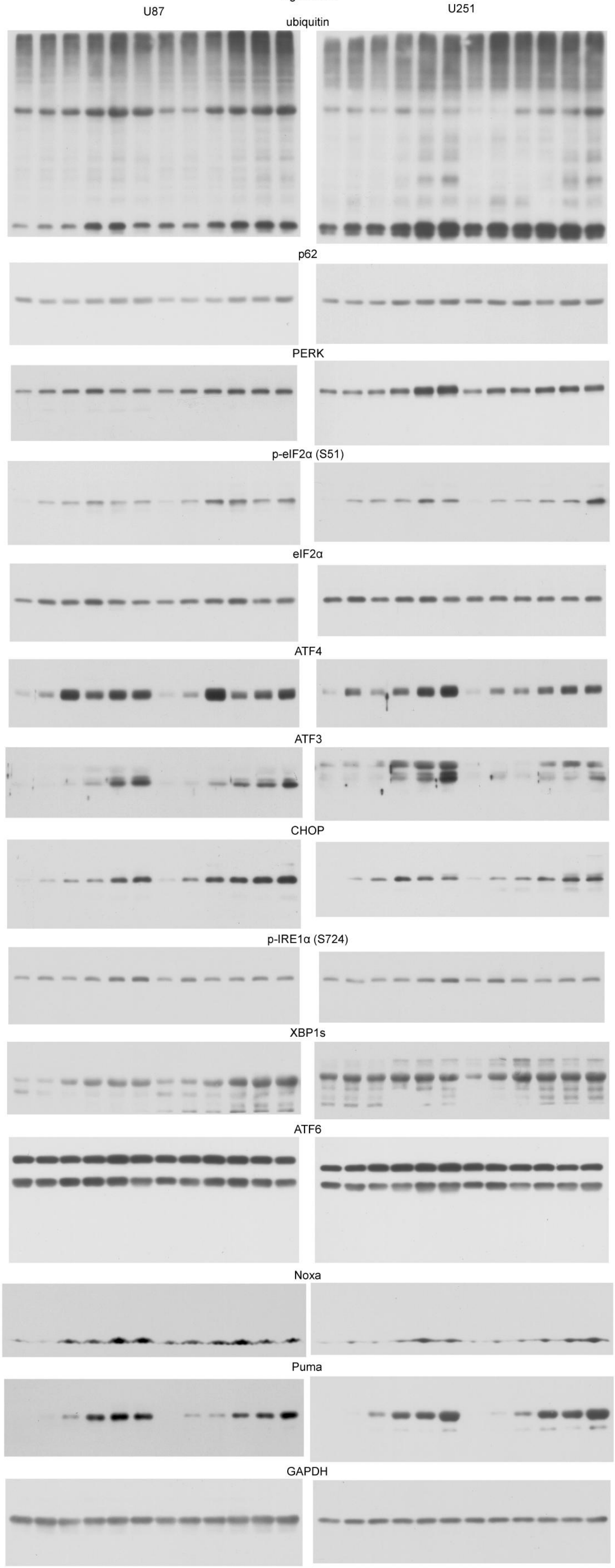

Figure S5f

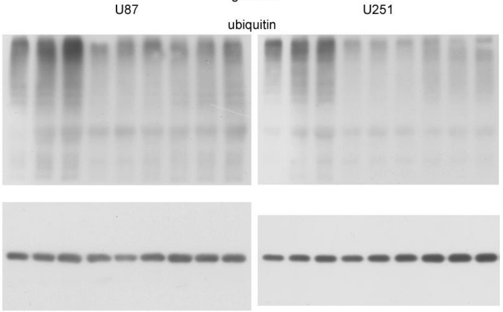

Figure S5g

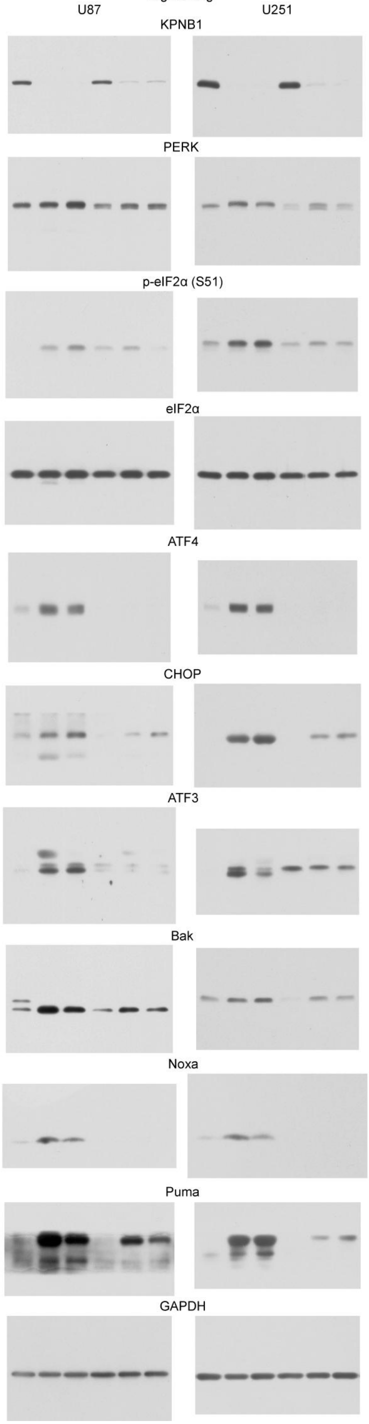

Figure S5i

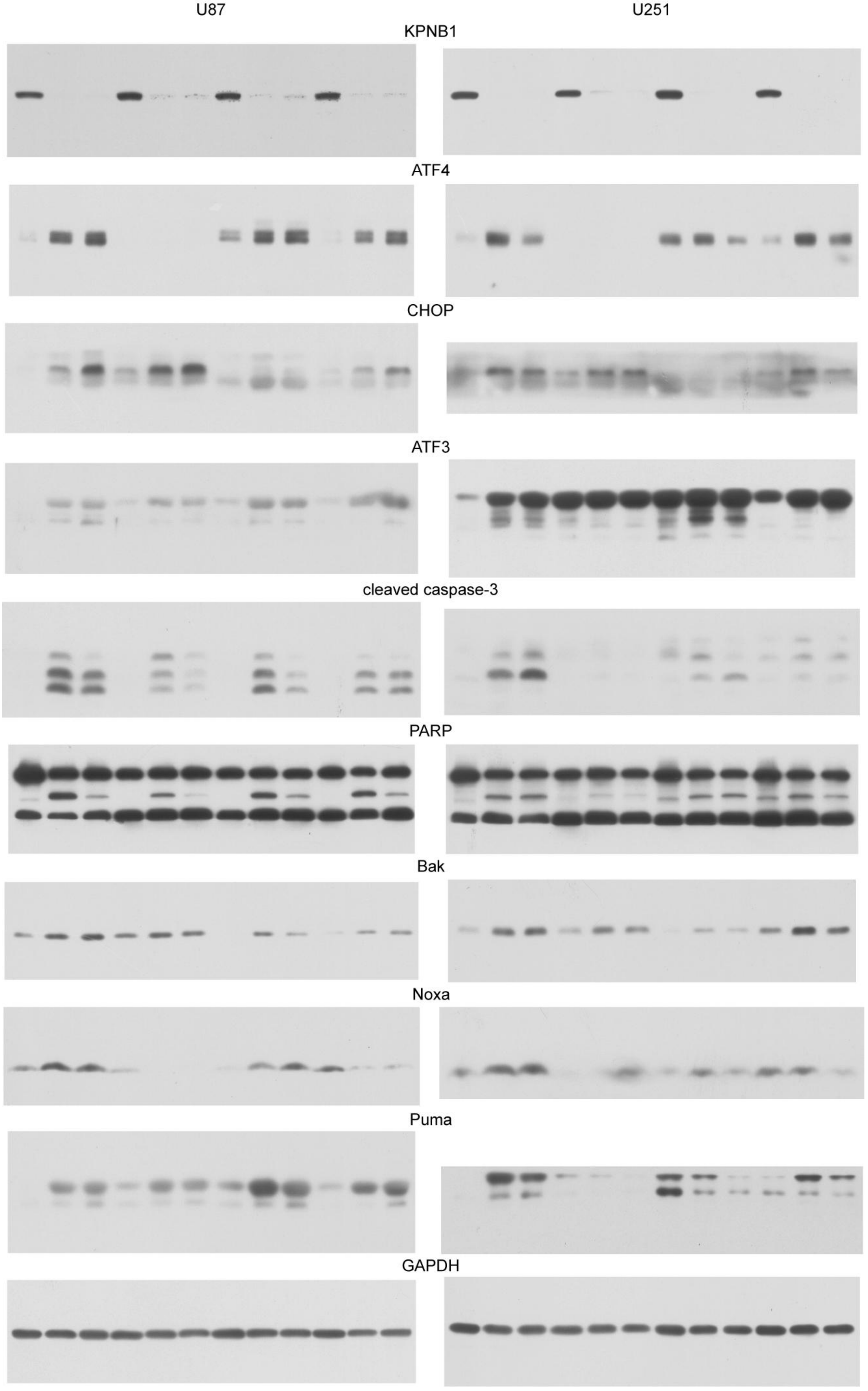
U87
U251
KPNB1
ATF4
CHOP
ATF3
cleaved caspase-3
PARP
Bak
Noxa
Puma
GAPDH
